# Halogen Bonds of Halogen(I) Ions—Where Are We and Where to Go?

**DOI:** 10.1021/jacs.3c11449

**Published:** 2023-12-20

**Authors:** Lianne H. E. Wieske, Mate Erdelyi

**Affiliations:** Department of Chemistry−BMC, Uppsala University, Box 576, SE-751 23 Uppsala, Sweden

## Abstract

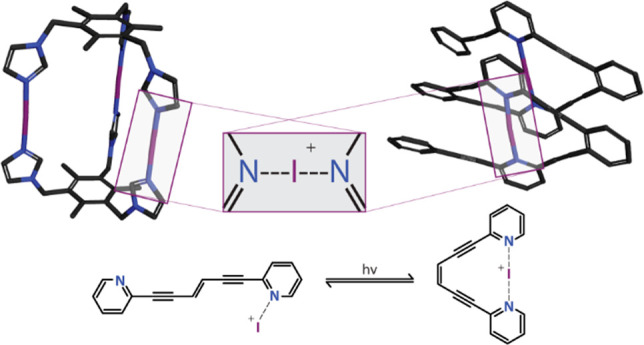

Halenium ions, X^+^, are particularly strong halogen-bond donors that interact with two Lewis bases simultaneously to form linear [D···X···D]^+^-type halonium complexes. Their three-center, four-electron halogen bond is both fundamentally interesting and technologically valuable as it tames the reactivity of halogen(I) ions, opening up new horizons in a variety of fields including synthetic organic and supramolecular chemistry. Understanding this bonding situation enables the development of improved halogen(I) transfer reactions and of advanced functional materials. Following a decade of investigations of basic principles, the range of applications is now rapidly widening. In this Perspective, we assess the status of the field and identify its key advances and the main bottlenecks. Clearing common misunderstandings that may hinder future progress, we aim to inspire and direct future research efforts.

## Introduction

**Figure 1:**
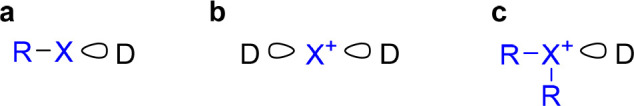
Halogen bond of (a) conventional monovalent halogen-bond donors, (b) divalent halogen(I) ions, and (c) trivalent λ^3^-halogane ions. The electrophilic moiety is the antibonding orbital of a carbon–halogen σ-bond, a σ-hole, for the mono- and trivalent halogen-bond donors, whereas it is an empty p orbital, a p hole, for divalent halogen(I) ions. D stands for the Lewis base and R for any alkyl or aryl group.

**Figure 2:**
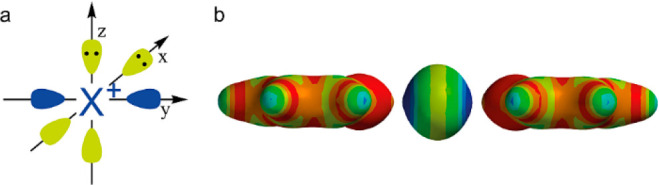
(a) The empty p orbital (blue) of the halenium ions forms two p holes, opposing each other and separated by a neutral equator (yellow). (b) Each p hole may interact with a Lewis base, here with the nonbonding electron pairs of a pyridine nitrogen, forming a halonium complex. The surface electron density is visualized with red for the most electron-rich and blue for the most electron-poor regions.

Halogen bonding^1^ is a noncovalent interaction that has for a long time been overlooked^2^ but subsequently has left a stamp on virtually all fields of chemistry.^3^ Following a series of fundamental studies, it has found applications, among others, in crystal engineering,^4,5^ medicinal chemistry,^6,7^ material sciences,^8,9^ and organic synthesis.^10^ Halogen bonding resembles hydrogen bonding to a great extent^11^ and by analogy is defined as the attractive interaction of an electrophilic region of a halogen with a Lewis base. It is directional,^12^ and its strength is modulated by the electron density and type of halogen involved. As more electron-poor halogens give stronger halogen bonds, halenium ions (X^+^) that carry a full positive charge evidently form stronger interactions (up to 180 kJ/mol) than neutral halogens that are covalently bound, for instance, to a carbon (typically <20 kJ/mol).^13^ Covalently bound halogens, including λ^3^-haloganes, dihalogens, as well as alkyl and aryl halides, act as halogen-bond donors due to the electrophilicity of their C–X antibonding orbital. This is the noninvolved electron-deficient lobe of a half-filled p orbital of the halogen that participates in forming a covalent bond to a next atom (R in Figure 1).^14^ In contrast, halenium ions establish halogen bonds through their empty p orbital. As this has two lobes, it prefers to interact with two Lewis bases simultaneously (Figure 2) and forms a three-center, four-electron (3c4e) bond,^15−18^ [D···X···D], which is remarkably strong (where D is a Lewis base and X a halogen). Importantly, a three-center halogen bond forms for all types of halogens but for fluorine(I)^19^ and for any type of Lewis base (N, O, S, X, etc) involved.^13,17^

This interaction has dominantly charge transfer and electrostatic character.^19,20^ Halogen-bonded halenium ions, [D···X···D], are halogen(I) species that are frequently termed halonium ions.

Halogen-bonded halogen(I) ions have been known and been in use for decades^21,22^ but for a long time without an understanding of the nature of their bonding. The triiodide ion, [I^–^···I^+^···I^–^], [I–I···I^–^], or [I···I···I]^−^, commonly depicted as I_3_^–^, has been known for two centuries, whereas bis(pyridine)iodonium tetrafluoroborate was marketed by Barluenga as an iodination and oxidation agent in the 1980s.^23,24^ The halogen bond of these complexes has first been discussed in 2012^15,16^ and ever since been intensely studied and repeatedly reviewed.^13,17,25−28^ A decade of fundamental spectroscopic, crystallographic, and computational investigations was followed by an array of initial applications in, for instance, supramolecular chemistry and organic synthesis with a growing number of research groups working on the topic. In this Perspective, we assess the current status of the field, identifying its key advances as well as its main bottlenecks. We aim not only to inspire and direct future research efforts but also to clear misunderstandings that may hinder future progress. Accordingly, we dedicate sections to topics that need to be addressed to maintain quality, which we see as essential for further development of this flourishing research field.

## Terminology

A variety of terms are in use to describe halogen-bonded halogen(I) complexes. Some may have a different meaning to different people; others, despite sounding evocative, may be misleading. The use of parallel nomenclatures may lead to the rediscussion of already studied concepts, under new names, which is unproductive. Conversion to a commonly accepted, clear terminology is expected to facilitate a productive and coherent scientific discourse. This we wish to support by discussing the terms that are currently in use and recommending a notion for the future literature.

Halonium ions are open-chain or cyclic onium ions of the form D_2_X^+^, where X is a halogen.^29^ Halonium ions bound to two carbons, [C–X–C]^+^, do not possess halogen-bond donor character and were comprehensively explored by Kimball,^30^ Olah,^31,32^ Wyndberg,^33^ Brown,^34^ Nugent,^35^ Kochi,^36^ Denmark,^37^ and others. When nitrogen, sulfur, oxygen, or halogen-donor Lewis bases (D) are involved, [D···X···D]^+^ halogen-bond complexes are formed. Here, [D···X···D]^+^ denotes the hypervalent/hypercoordinate halonium ion, which is the halogen-bonded halogen(I) complex, and the halogen-bond donor Lewis acid X^+^ is a hypovalent/hypocoordinate halenium ion.^13^ This ion may also be described as a 10-X-2 complex, as its halogen(I), X, possesses 10 valence electrons and binds to 2 ligands. Four of the electrons are donated by the two Lewis bases, whereas six originate from the hypovalent halenium ion, X^+^. As three atoms are held together by four electrons and the interaction requires the presence of all three, the bond is classified as a three-center, four-electron (3c4e) halogen bond, following the Pimentel–Rundle theory.^38−40^ The D···X halogen-bond distances are longer than a covalent D–X bond and have partial covalent and partial electrostatic character.^13,19^ These bonds have sometimes been referred to as “coordinative halogen bonds” or “halogen bonds with coordinative nature”,^41,42^ which are terms that are misleading and should be avoided. Halogen(I) complexes do not behave as coordinative transition metal complexes,^43^ for example, when exposed to solvents and counterions.^44^ The bond has occasionally also been named a halonium bond^45^ and the complexes superhalides.^21,22^ We note that halogen(I) complexes should not be misconceived with λ^3^-halogane (λ^3^-iodane, λ^3^-bromane, λ^3^-chlorane) compounds, which sometimes are also termed halonium complexes yet encompass a halogen(III).^46,47^

In a 3c4e halogen bond, [D···X···D]^+^, the positive charge of the halenium ion, X^+^, is transferred to a large extent to the coordinating Lewis bases (D),^13,17,19^ and therefore, we recommend the use of the term “halogen(I) complex” as it was initially introduced by Lin and Hope in 1972.^48^ This provides a more accurate description of the charge distribution than the term “halonium complex”, which in turn presumes the positive charge to be located on the halogen, which, however, is not the case. To emphasize the charge transfer character of these complexes,^20^ on the recommendation of Rissanen,^26^ halogen-bonded halogen(I) complexes are described as [bis(ligand)halogen(I)]^+^ rather than bis(ligand)halonium species (that is [bis(pyridine)iodine(I)]^+^ instead of bis(pyridine)iodonium complexes). We recommend the continued use of this notion.^13,27^

The D···X bonds of halogen(I) complexes are in most cases comparable in the bond length. Such complexes are designated as symmetric,^15,16^ whereas those possessing different Lewis bases, D, having slightly different D···X bond lengths while retaining their 3c4e character are termed asymmetric.^49^ These geometries have recently been remarketed as homo- versus heteroleptic.^50,51^ As there is no conceptual difference of the latter nomenclature to the original notion, our recommendation is to retain the terms symmetric and asymmetric to avoid the creation of parallel discussions of the same phenomenon.

## Synthesis

**Scheme 1:**
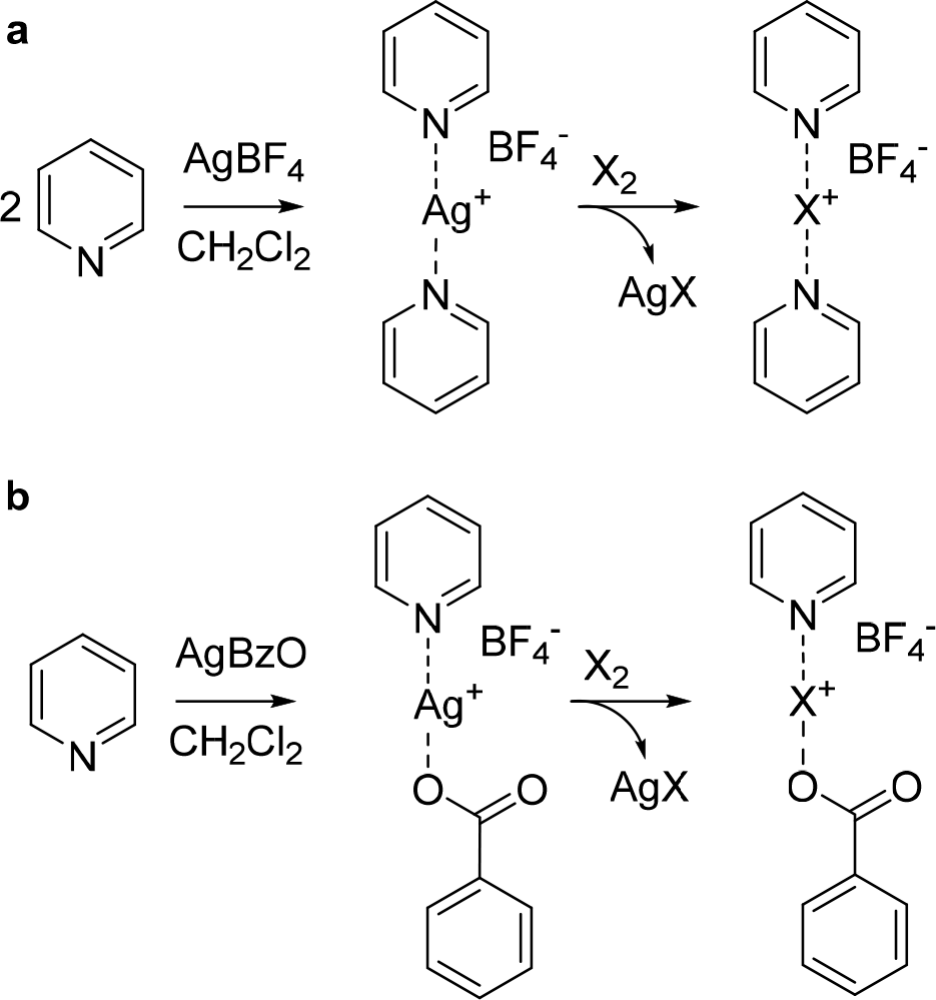
Halogen-Bonded Halogen(I) Complexes Are Conventionally Generated from Their Silver(I) Analogues Using X_2_ = I_2_, Br_2_, or Cl_2_^,^^16,19^ Tetrafluoroborate is one of the most common counterions for [bis(pyridine)halogen(I)]^+^ complexes.^44^

Halogen-bonded halogen(I) complexes are conventionally prepared from the analogous silver(I) complexes (Scheme 1) by addition of molecular halogen, X_2_. The driving force of the reaction is the precipitation of silver halide, which may be removed either by centrifugation or on larger scale by filtration. Subsequently, the halogen(I) complex is isolated by precipitation using nonpolar solvents, such as pentanes or hexanes.^15,52^ This protocol is typically performed in CH_2_Cl_2_ but also works using other aprotic solvents, such as CH_3_CN.^53^ Being a robust procedure, it can be applied for the preparation of halogen(I) complexes using various Lewis bases to yield virtually any type of [D···X···D] complexes, independent of charge and donor atom, thus including, for instance, the [O–X–O]^+^ ^54^ and [N–I–O]^55,56^ analogues. Importantly, this procedure is unlikely to provide pure product in the presence of protic solvents, such as alcohols or water, which have been used in some initial protocols.^21,57,58^ In protic solvents, the protonated Lewis base, or the halogen(I) complex contaminated with it, is formed according to D_2_I^+^ + H_2_O → 2DH^+^ + OI^–^, where D is the Lewis base. Due to the high electrophilicity of halogen(I), these complexes rapidly react with moisture when not properly dried solvents and glassware are used. This instability was reported early on^58^ but is sometimes forgotten. The halogen(I) complex may be isolated from such mixtures by recrystallization, as described by Hope and Hassel;^58^ however, the solution will at best contain a mixture of product and byproduct. As the [D_2_H]^+^ and [D_2_I]^+^ complexes may be in rapid chemical exchange, only a single set of NMR signals might be detected, due to signal coalescence, which can lead to unfortunate data misinterpretations.^60,61^ The ^1^H and ^13^C NMR chemical shifts of [bis(pyridine)iodine(I)]^+^ and protonated pyridine, for instance, are similar and hence are not sufficient for distinguishing these species. The ^15^N NMR chemical shifts, acquired via ^1^H,^15^N HMBC, can best be used for the analysis of halogen(I) complexes. Reference ^15^N NMR chemical shifts for bis(pyridine)halogen(I) complexes and the corresponding protonated and silver(I) complex are available (Table 1).^15,19,62,63^ We see the use of properly dried aprotic solvents accompanied by careful NMR characterization of the resulting complexes as a key for the progress of the field. This will help to avoid unfortunate misapprehensions. We wish to further note that halogen(I) complexes can be synthesized in the presence of a wide variety of counterions,^44^ but the hygroscopicity of the resulting salts is counterion dependent and has a noticeable influence on the stability of the complex. The use of weakly coordinating spherical anions, such as BF_4_^–^, PF_6_^–^, and SbF_6_^–^, is preferred over strongly coordinating ones, such as OTF^–^, CF_3_COO^–^, and OAc^–^, to achieve less hygroscopic complexes.

**Table 1:** ^15^N NMR Chemical and Coordination Shifts^a^ (ppm) of the Halogen(I), Silver(I), and Proton Complexes of Pyridine^15,19,62,63^

entry	structure	δ(^15^N)	δ(^15^N)_coord_
1	pyridine (Pyr)	–67.0	
2	PyrH^+^BF_4_^–^	–186.5	–119.5
3	(Pyr)_2_H^+^BF_4_^–^	–134.1	–67.1
4	(Pyr)_2_Ag^+^BF_4_^–^	–126.5	–59.5
5	(Pyr)_2_I^+^BF_4_^–^	–175.1	–108.1
6	(Pyr)_2_Br^+^BF_4_^–^	–142.9	–75.9
7	(Pyr)_2_Cl^+^BF_4_^–^	na^c^	na^c^
8	(Pyr)_2_F^+^BF_4_^–^	–122.1 /–68.8^b^	–55.1/–1.8
9	(4-NMe_2_-Pyr)_2_I^+^BF_4_^–^	–214.2	–104.8
10	(4-CF_3_-Pyr)_2_I^+^BF_4_^–^	–164.1	–112.5

a The ^15^N NMR coordination shift represents the chemical shift change upon complex formation, δ(^15^N)_coord_ = δ(^15^N)_complex_ – δ(^15^N)_ligand_.

b Measured in CDCN_3_ at −35 °C; all other chemical shifts given here were measured in CD_2_Cl_2_. The fluorine(I) complex is asymmetric, and accordingly, its nitrogens have different chemical shifts.

c Due to the instability of this complex,^19,64^ its ^15^N NMR chemical shift data is unavailable.

**Scheme 2:**
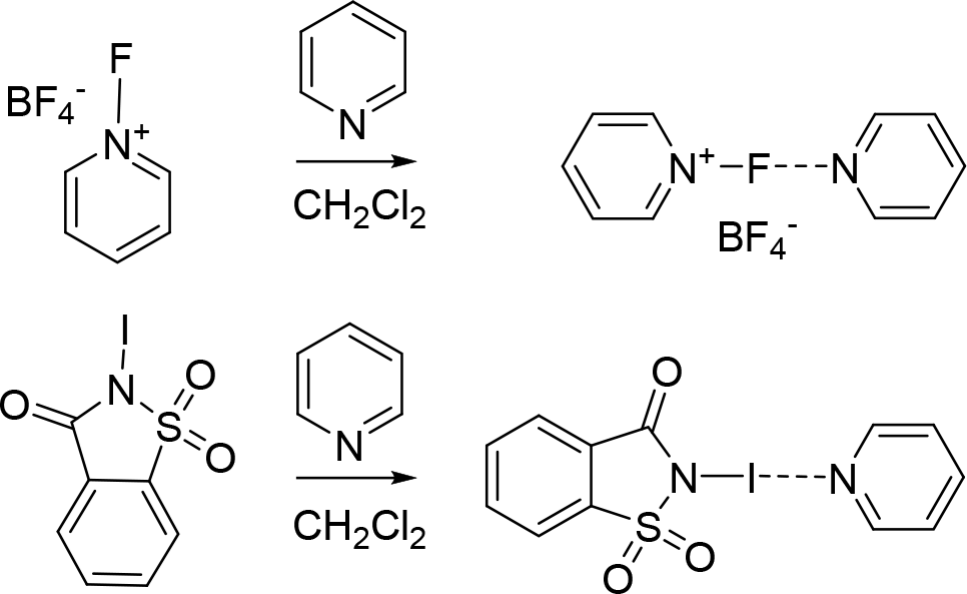
Generation of Halogen-Bonded Halogen(I) Complexes by Mixing an *N*-Halogenated Lewis Base with Another Lewis Base^,^^19,50,59^ It is important to remember that the presence of two or more Lewis bases yields a mixture of halogen-bond complexes (Scheme 3).

An alternative option for the generation of halogen-bonded halogen(I) complexes is to mix stabilized *N*-halonium compounds, such as *N*-fluoropyridinium salts^19^ or *N*-haloimides,^59^ with a Lewis base (Scheme 2). This as a rule gives asymmetric complexes in which one of the bonds to halogen(I) has a stronger covalent character than the second weaker noncovalent bond. Such complexes may not have a true 3-center-4-electron bond character but may resemble conventional halogen bonds and are then accordingly weak.^19^ It is important to note that in cases where two different Lewis bases are present in solution, a mixture of complexes is formed as a rule with their population being dependent on the binding constants and the relative concentrations.

A third alternative to generate halogen-bonded halogen(I) complexes makes use of ligand exchange with a stronger Lewis base in an excess. As halogen bonding is a noncovalent interaction, halogen(I) complexes are present in solution as dynamic mixtures of quickly associating and dissociating species (*K*_d_ ≈ 0.9).^65,66^ Mixing two or more dissimilar Lewis bases with comparable nucleophilicity in the presence of halogen(I) leads to the formation of a mixture of complexes. The most polar asymmetric form can occasionally be isolated from such mixtures by crystallization.^51^ Generating a mixture of products, this technique is not very useful for the synthesis of complexes for fundamental studies. We expect, however, that this method will gain importance in the production of insoluble halogen(I) complexes, such as halogen-bonded frameworks (XOFs), as it provides a halogen(I) complex without silver halide precipitate as a byproduct. At the production of halogen-bonded frameworks, this is a major advantage since coprecipitation of silver halide contaminates the framework, and there are at best very limited possibilities, if any, for the separation of the insoluble coprecipitates.^57^ Thus, we recommend the exploration of this synthetic route especially for upcoming studies of halogen-bonded frameworks.

A further option for the formation of halogen(I) complexes is to mix a strong Lewis base, such as DMAP or a thione, with a dihalogen to generate a halogen(I) complex.^42^ This reaction was used in the very first reports on the structural investigation of halogen(I) complexes by Prescott and Trowbridge in 1895^21^ as well as by Hope and Hassel in 1961,^58^ whose impact on this research field cannot be overstated.^20^ This redox reaction does not generate a pure product in high yield, but the product can be isolated through careful crystallization. Riedel and co-workers have used an analogous strategy for the production of [bis(pyridine)chlorine(I)]^+^ and bis(luthidine)chlorine(I)]^+^Cl_3_^–^ crystals from −196 to −40 °C,^64^ which has been a truly impressive achievement considering that such a complex could previously only be studied in solution at −80 °C.^19,67^

In summary, halogen-bonded halogen(I) complexes can be produced through a variety of robust synthetic routes; however, one needs to carefully exclude moisture and avoid the use of protic solvents to obtain pure products. For their characterization in solution, the detection of ^15^N NMR chemical shifts is strongly recommended as the ^1^H and ^13^C NMR spectra of the halogen(I) and proton complexes are often similar.

## Fundamentals

The basic principles of the bonding of halogen(I) complexes have initially been explored by X-ray diffraction,^48,58,68^ Raman spectroscopy,^69^ and calculation.^70^ These were followed by scarce reports based on X-ray diffractometric observations over the period of 1970–2010.^68,70−76^ The field has received increasing interest over the past decade, leading to the generation of a rapidly growing pool of experimental and theoretical data. Presently, the most knowledge is available for [bis(pyridine)halogen(I)]^+^ complexes, studies that have provided the basis of our current understanding. It should be emphasized that the nature of the halogen bond and the properties of halogen(I) complexes are largely independent of the overall charge of the complex ([D···X···D], [D···X···D]^+^, or [D···X···D] ^–^) and of the type of Lewis base (N, O, S, X) involved in complex formation.

### Three-Center Bond Character

**Figure 3:**
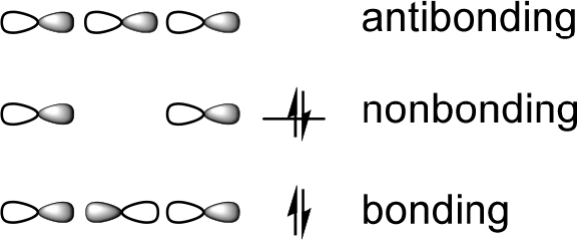
Molecular orbital diagram for the 3c4e halogen bond of halogen(I) ions I^+^, Br^+^, and Cl^+^. Two of the four electrons are on the bonding and two on the nonbonding level.

**Figure 4:**
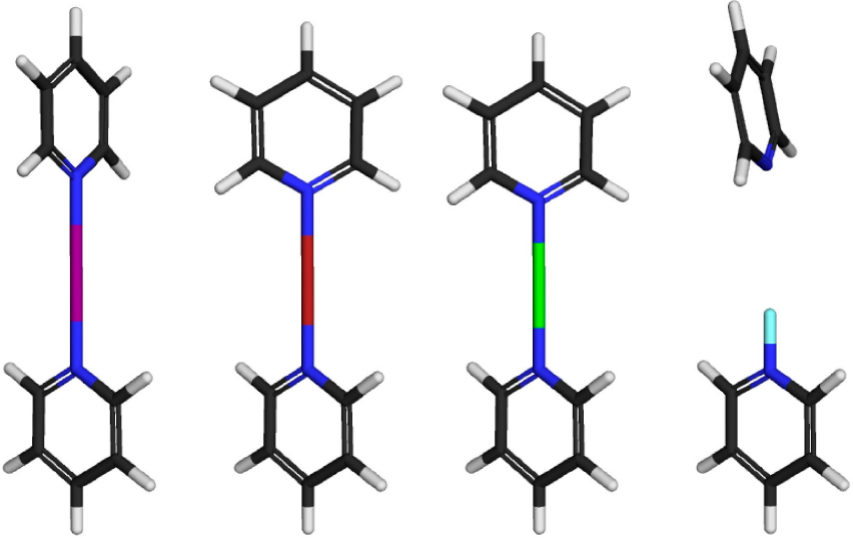
DFT geometry-optimized (B3LYP/LANL08d) structure of [bis(pyridine)halogen(I)] complexes. From left to right: [N···I···N]^+^, [N···Br···N]^+^, [N···Cl···N]^+^, and [N–F···N]^+^.^19^

In the electrostatic field of two Lewis bases, the p orbitals of halogen(I) are occupied in the spin-paired p_*x*_^2^p_*y*_^2^p_*z*_^0^ arrangement (Figure 3).^13,17,48,53^ The two empty lobes of the p_*z*_^0^ orbital interact with the electron pairs of two Lewis bases, D, resulting in formation of a linear (180°) [D···X···D] bond. Depending on whether the Lewis bases are neutral or anionic, the halogen-bond complex becomes cationic,^16^ neutral,^55^ or anionic.^50^ Independently of the charge, a strong three-center bond is formed. This bond has covalent (charge transfer)^20^ and electrostatic (Coulomb attraction) character, whereas the contribution of dispersion is negligible.^19^ The covalent nature of the bond increases as the size of the halogen decreases. The strength of the bond increases in the order Cl < Br < I, as it mainly originates from the electrostatic character.^13,19^ Still, a simple electrostatic model is unable to explain the stability of three-center halogen-bond complexes.^45^ These halogen bonds are characteristically short and strong, Δ*G* > 50 kJ/mol, with their bond strength and length depending on the involved halogen and Lewis bases. The bond distance is often characterized by the reduction of the contact distance of the interacting atoms in comparison to the sum of their van der Waals radii, which for three-center bonds is 60–70% (*R*_XB_ = 0.6–0.7).^13,26^ The bond lengths and the bond strengths decrease with the size of the involved halogen(I) decreasing. For [bis(pyridine)halogen(I)]^+^ complexes, the N–X bond length is ∼2.3 Å for [N···I···N]^+^ (*R*_XB_ = 0.65), 2.1 Å for [N···Br···N]^+^ (*R*_XB_ = 0.63), and 2.0 Å for [N···Cl···N]^+^ (*R*_XB_ = 0.61) complexes, whereas it is 1.4 and 3.5 Å for the [N–F···N]^+^ complex (Figure 4). The fluorine(I)-centered complexes are best described as [D–F]^+^···D interactions, with a short and strong covalent N^+^–F and a long and weak N···F halogen bond.^19,45^ Initially, even fluorine(I) complexes have been predicted to be symmetric,^70^ a suggestion that was later experimentally and computationally refuted.^19,41^ The bond energies strongly depend on the Lewis basicity of the electron donors involved. The bond length is, in contrast, largely electron density independent. Accordingly, a 87 kJ/mol variation in bond energy is associated with a 0.0011 Å variation in bond length, as shown using substituted [bis(pyridine)iodine(I)] complexes.^13,62^ Such a small alteration in bond length is unmeasurable with current X-ray diffractometers. Conclusions on the bond strength of three-centered halogen bonds based on interatomic distances measured by X-ray diffraction are therefore often misleading. The halogen bond lengths of halogen(I)s bound to different Lewis bases are dominated by the properties of the electron-donor atoms and hence are naturally different for complexes involving O, N, and S donors or for aliphatic and aromatic N donors, independent of the bond strength of the halogen bond formed.

### Symmetry

**Figure 5:**
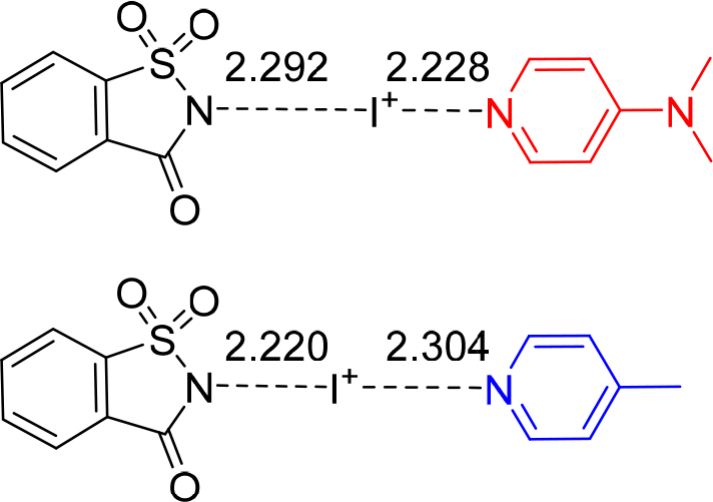
N–I bond distances (Ångströms) of the halogen-bonded complexes of *N*-iodosaccharin and (a) 4-(dimethylamino)pyridine and (b) 4-methylpyridine, as observed by X-ray crystallography.^83^

The position of the halogen(I) within the three-center bond and the factors possibly affecting it have been extensively studied over the past decade. These halogen bonds strongly prefer a linear and symmetric arrangement as confirmed by computation,^15,17,18^ NMR,^15,16^ and single-crystal X-ray diffraction.^18,25,44,63,64,72^ The symmetric arrangement remains independent of the solvent polarity,^53^ the identity of the counterion,^44^ the electron density of the Lewis bases,^50,51,55,62^ and weak steric and electronic effects.^77^ The outcome of the initial studies have been corroborated by further computational^45,78−80^ and single-crystal X-ray diffraction^50,63^ investigations. Halogen-bond symmetry may be broken using electron donors with different Lewis basicities^49,59,81,82^ or through secondary interactions to only one of the two halogen-bonded Lewis bases.^50^ If the Lewis basicities of the two coordinating electron donors do not differ drastically, slightly asymmetric complexes with a strong 3c4e bond are formed.^49^ In such a complex, the halogen(I) is positioned closer to the more Lewis basic coordinating ligand (Figure 5).^83^ It is important to remember that halogen(I) complexes of monodentate ligands easily dissociate in solution, resulting in ligand scrambling. The asymmetric complex may by chance be isolated by crystallization, whereas in solution, the ligands rapidly interchange (scramble), yielding a mixture of symmetric and asymmetric species.^65^ Studying the bond of (a)symmetric halogen(I) complexes using a bidentate ligand,^15,44,49,62,77^ which prevents ligand scrambling, lowers the risks of misinterpretations.

It has been predicted that asymmetric complexes may be prepared by enforcing a longer than optimal distance between the coordinating Lewis bases.^49^ In practice, such attempts led to formation of symmetric halogen-bonded dimeric complexes of the bidentate ligands rather than asymmetric monomers. Forming a stable asymmetric complex with a longer than optimal Lewis base distance remains an unsolved challenge.

The concept of halogen-bond symmetry is closely associated to the ability of controlling halogen(I) transfer between Lewis bases.^49^ Hence, the [D–X···D]^+^, [D···X···D]^+^, and [D···X–D]^+^ geometries represent varying stages of a halogen(I) ion transfer from one halogen-bond acceptor to another one. These stages show a varying degree of covalency and D–X bond length. Understanding whether a halogen transfer in between two halogen-bond acceptors follows a single-well or double-well potential and whether a symmetric geometry is energetically favorable relative to a resonance-stabilized equilibrating mixture of asymmetric ones (halotropy) is of fundamental importance and accordingly has raised wide interest.^15,16,25,49,59,81,83−85^ It should further be noted that electrophilic halofunctionalizations follow an analogous halogen(I) transfer process: (i) the initial weakening of a covalent bond of a halogen; (ii) formation of a 3c4e halogen-bond complex consisting of a nucleophile, a halogen(I), and a leaving group; (iii) collapse of this intermediate upon formation of a covalent halogen–nucleophile bond and leaving group elimination. Accordingly, understanding halogen-bond symmetry is a key step toward the development of (enantioselective) halogen(I) transfer strategies.

### Halotropy

The similarities of halogen and hydrogen bonds have been often emphasized,^11^ in which context we wish to stress that the symmetry of H^+^ and X^+^ complexes is fundamentally unalike. Three-center hydrogen bonds are neither short nor strong, and such complexes are present in solution as a mixture of rapidly interconverting asymmetric geometries.^86,87^ In contrast, when studied with the same techniques, the corresponding three-center halogen bonds of iodine(I), bromine(I), and chlorine(I) are symmetric, short, and strong.^13,17,25^ This difference presumably originates in the size and electron affinity of hydrogen(I) and halogen(I) and hence the orbitals involved in the interactions.^18^ The empty s orbital of H^+^ is small and unable to simultaneously interact with two nonbonding/p orbitals, while such concomitant interaction is favorable for the larger and directional empty p orbital of halogen(I). Accordingly, halogen(I) complexes do not show halotropy, in contrast to the prototropy, [D–H···D]^+^ ↔ [D···H–D] ^+^, of the analogous [D···H···D]^+^ systems.

### Dynamics

**Scheme 3:**
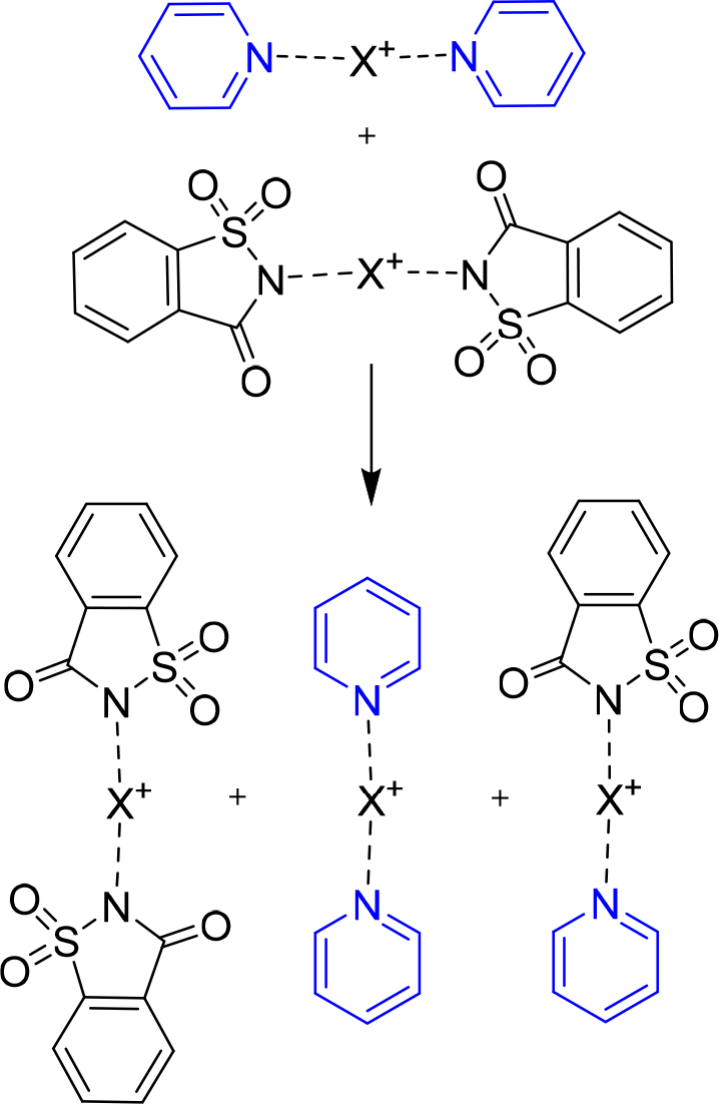
Similar to Other Chemical Entities Held Together by Noncovalent Forces, Halogen-Bonded Halogen(I) Complexes Undergo Quick Dissociation–Associate Equilibria in Solution Consequently, if more than one Lewis base is present in solution, a rapid ligand exchange (scrambling) takes place.

Halogen(I) ions’ halogen bonds are unusually strong, and therefore, the consequences of their noncovalent character are occasionally neglected, presuming these interactions to be as static as covalent bonds. Halogen(I) complexes are, however, present in solution as dynamic mixtures of their associated and dissociated forms (Scheme 3). Presuming a bond strength of Δ*G*° ≈ 150 kJ/mol, the equilibrium constant is *K* ≈ 0.94 M (Δ*G*° = −*RT* ln *K*), which corresponds to 3% dissociation in a 100 mM and 10% dissociation in a 10 mM solution (α = √(*K*/*c*), where *c* is the concentration and α is the degree of dissociation). Accordingly, if two or more possible ligands with reasonably similar Lewis basicity (Δp*K*_a_ < 10) are present in solution, the mixture rapidly equilibrates and complexes encompassing all possible combinations of Lewis bases are present in solution.^65,66^ Consequently, forming a halogen(I) complex with two different monodentate Lewis bases does not lead to formation of an asymmetric (heteroleptic) complex, [D_1_–X–D_2_], in solution but to a mixture of all possible species, [D_1_–X–D_2_], [D_1_–X–D_1_], and [D_2_–X–D_2_], with their populations being dependent on the association constants and relative concentrations.^51,65^ Moreover, a halogen(I) complex cannot be converted into another complex via simple ligand exchange in solution, with the exception of special cases, for instance, when the new product selectively precipitates and thereby leaves the equilibrium. This might be useful for the generation of halogen-bonded frameworks, avoiding silver halide contamination by coprecipitation. It should also be emphasized that the ligand exchange between halogen(I) and metal or proton complexes is rapid as well. A small amount of moisture consequently leads to mixtures of hydrogen- and halogen-bonded complexes in rapid exchange, which may be difficult to identify.^60^ There is an often neglected ligand exchange between silver(I) and halogen(I) complexes in solution, when silver(I) ions remain due to incomplete silver(I) to iodine(I) exchange at the preparation of halogen-bond complexes. Lewis basic solvents and counterions of comparable Lewis basicity to that of the Lewis base of the complex may also compete for binding to the halogen(I). We see that neglecting the dynamic nature of the halogen(I) complexes and the consequent equilibrium processes are a major risk for the progress of the research field.

### Lewis Base

[Bis(pyridine)halogen(I)] complexes have by far been the best studied,^15,16,23,26,27,44,50,51,60−63,65,88−91^ yet a number of analogues with other nitrogen,^50,59^ oxygen,^54,92^ sulfur,^42,68,76,93−95^ selenium,^73,75,76,96,97^ and tellurium^98^ donor ligands as well as complexes with mixed nitrogen/oxygen donor Lewis bases^81^ have also been reported. Stronger Lewis bases (O^–^ > N > O > S > Se > Te) form complexes with stronger halogen bonds.^54^ The [O···I···O]^+^ complexes of pyridine *N*-oxides are linear and centrosymmetric and slightly, ∼10 kJ/mol, stronger than the analogous [N···I···N]^+^ complexes.^54^ They are, nonetheless, more moisture sensitive, which explains the lack of reported X-ray structures. Crystal structures are available for the [O···I···O]^−^ complex^92,99,100^ and corroborate the linear and centrosymmetric arrangement (*d*_IO_ = 2.195 Å, *R*_XB_ = 0.63), typical for 3c4e halogen bonds. The anionic [N···I···N]^−^ and [N···Br···N]^−^ complexes are also linear and possess near identical N–X bond lengths to the analogous [N···I···N]^+^ and [N···Br···N]^+^ complexes, indicating that this halogen bond is insensitive to the type and charge of the Lewis bases involved.^50^ In contrast to [N···X···N]^+^ complexes, weaker [S···X···S]^+^ complexes have so far only been reported for iodine(I) by X-ray studies and have not yet been observed in solution.^42,68,76,93−95^ The S–I halogen-bond distances vary more (2.59–2.70 Å)^28^ than the N–I distances (4.66–4.77 Å)^62^ of the corresponding complexes do. As a result of the lower nucleophilicity of sulfur, the average *R*_XB_ is 0.70,^28,42^ indicating slightly longer halogen bonds than the *R*_XB_ = 0.65 reported for [N···I···N]^+^ complexes.^19^ [S···X···S]^+^ complexes also prefer a symmetric 3c4e halogen-bond geometry. There are only a few known [Se···X···Se]^+^ ^73,75,76,96,97^ and [Te···X···Te]^+^ ^98^ complexes, most of them having iodine(I) as the halogen-bond donor,^28^ and average *R*_XB_ = 0.72. The S-, Te-, and Se-based halogen(I) complexes have only been studied in the solid state by X-ray diffraction, not yet in solution.

Complexes with mixed nitrogen/oxygen donor Lewis bases have been studied over the recent years.^81,82^ The [N···I···O]^−^ halogen-bond complex encompassing *N*-iodosuccinimide and acetate as Lewis bases has been crystallized and showed a linear geometry with *d*_NI_ = 2.17 Å (*R*_XB_ = 0.61) and *d*_OI_ = 2.30 Å (*R*_XB_ = 0.66).^101^ Similar linear [N···I···O]^−^ interactions with shorter N–I than I–O bonds were reported for the iodine(I) complexes of *N*-iodosaccharins and pyridine.^102^ The linear [N···I···O]^−^ halogen bonds formed upon the interaction of *N*-iodosuccinimide or *N*-iodosaccharin with pyridine *N*-oxide have short N–I (2.14 Å, *R*_XB_ = 0.60) and O–I (2.32 Å, *R*_XB_ = 0.66) distances.^81,82^ The interaction energy was estimated to be up to 120 kJ/mol in CDCl_3_ by measuring the association constants by NMR titrations (*K*_a_ up to 10^8^ M). Analogous bromine(I)-centered [N···Br···O] complexes have also been assessed and showed similar yet weaker bonds, a trend that fits that observed for the bis(pyridine) complexes of bromine(I) and iodine(I).^15^ Importantly, the N–Br and N–I bond lengths of substituted analogues vary insignificantly and are independent of the bond strength.^82^ This observation is also in line with the strength independence of the N–I bond lengths of [bis(pyridine)iodine(I)]^+^ complexes,^62^ highlighting the similarity of the bonds of halogen(I) complexes, independent of the Lewis base involved. Neutral [N···I···O] complexes encompassing substituted pyridines and carboxylic acids as Lewis bases show the expected linear N–I–O geometry with N–I (2.24 Å) and O–I (2.21 Å) bond distances analogous to the previously studied [N···I···N]^+^ and [O···I···O]^+^ complexes.^56^

The influence of substituents on the stability and the geometry of halogen(I) complexes has been extensively studied using solution NMR, X-ray diffraction, and computations.^45,62,63,80^ In short, electron-donating substituents strengthen while electron-withdrawing ones weaken the 3c4e halogen bond. It is important to note that the halogen-bond strength shows a strong dependence on the electron density of the Lewis base, whereas the halogen-bond donor–acceptor distance and the NMR coordination shifts do not show a larger strength-dependent variation.^62^

### Lack of Nucleophilicity

Despite their positive charge and filled electronic shells, cationic iodine(I) complexes, such as [bis(pyridine)iodine(I)]^+^BF_4_^–^, were proposed to be nucleophilic and establish an attractive interaction to cationic complexes.^61,103,104^ This hypothesis originated in the observation of short iodine(I)–silver(I) distances (3.40–3.52 Å, *R*_AgI_ = 0.92–0.95) in some X-ray structures and was complemented by computational, NMR, and calorimetric investigations. Follow-up work revealed that the ^15^N NMR chemical shift changes originally presented as proof for this interaction were the unfortunate result of neglected water contamination, ligand exchange between silver(I) and iodine(I) complexes, and the detection of low-resolution ^1^H,^15^N HMBC spectra preventing reliable detection of small chemical shift changes.^60^ Reinvestigation of the original computational data revealed that the interaction of the silver(I) and the iodine(I) of the studied complexes is endothermic (7–13 kJ/mol, depending on the computational method applied), where the repulsion is overcompensated by the attractive face-to-face π–π interaction of the electron-poor aromatic ligands (38–67 kJ/mol) bound to the cations. Despite this, the overall interaction of the cationic complexes remains much too weak to play a role or to be detectable in solution.^60^ The supplementary calorimetric evidence originally provided^104^ suggests an unexpectedly high binding constant (*K*_a_ ≈ 37 000 M^–1^, Δ*G* 21.6 kJ/mol) along with a positive entropy, +14.8 kJ/mol, upon formation of the complex, which is unanticipated for an association process. In our assessment, the interaction of iodine(I) and silver(I) is repulsive. In the studied systems, this repulsion is compensated by the π···π interaction of the electron-poor aromatic ligands of the complexes, resulting in an overall weak attractive force, which facilitates packing at crystallization but is undetectable in solution. An analogous short iodine(I)–iodine(I) contact has been reported earlier.^105^ The small Mayer bond order between the iodine(I) ions (BO = 0.01) indicates negligible orbital overlap. The proximity of the iodine(I) ions is permitted upon the redistribution of the charge from the iodine(I) ions to the conjugated π-system through charge transfer (halogen bonds),^20^ decreasing the Coulombic repulsion between the two cations. Our conclusion is that halogen(I) ions are not expected to act as nucleophiles.

### Stability

The stability of [D···X···D] complexes follows the I^+^ > Br^+^ > Cl^+^ trend, in general. Fluorine(I) does not form stable 3c4e complexes. Iodine(I) complexes can be stored for months as solids when dry, and they survive days in dry solutions in aprotic solvents. Bromine(I) complexes decompose within hours in solution, whereas chlorine(I) complexes can only be studied at low temperatures.^19,64,67^ The 3c4e halogen-bond complexes of electron-rich ligands are more stable those of electron-poor ones.^62^ The complexes of bidentate ligands, such as (1,2-bis(pyridin-2-ylethynyl)benzene), are comparably stable to their monodentate analogues, whether a symmetric or asymmetric halogen bond is formed.^15,49^ The most common cause of decomposition of [D···X···D] complexes is the presence of moisture or a protic solvent. Accordingly, halogen(I) complexes of 4-aminopyridine could be obtained when the complex was crystallized quickly, whereas a mixture of protonated and halogen(I) complexes was detected when a longer crystallization time was used.^106^ It is not uncommon that the analytical data presented to justify the existence of a halogen(I) complex corresponds to a protonated analogue.^107^

### NMR Characterization

The formation of halogen(I) complexes of N-donor ligands is best detected by the observation of ^15^N NMR coordination shifts. Due to the similarity of the ^1^H and ^13^C NMR chemical shifts of the free and complexed ligands, these are not suitable for detection or structural characterization. The analogous proton complexes that might be present as contaminants are sometimes mistaken for halogen(I) complexes, as they may have similar ^1^H and ^13^C NMR chemical shifts to those of the latter. The ^15^N NMR coordination shifts upon formation of [N···I···N]^+^ complexes are large (Table 1), importantly, characteristic for the type of Lewis base involved, and do not reflect the strength of the bond. Accordingly, the δ(^15^N)_coord_ of [bis(pyridine)iodine(I)]^+^ complexes is ca. −110 ppm, that of the analogous [(1,2-bis(pyridine-2-ylethynyl)benzene)iodine(I)]^+^ complexes is ca. −100 ppm,^62,77^ and that of the pyridine–hypoiodate complexes is ca. −105 ppm.^56^ It cannot be stressed enough that the magnitude of the coordination shifts within a structurally closely related series of complexes is independent of substitution, counterion, and solvent (Table 1, entries 5, 9, and 10).^13,44,53,62^ The coordination shifts are unaltered even in the absence of solvent.^108^ Variations <±5 ppm are typically the consequence of acquiring NMR chemical shifts with low resolution in the ^15^N dimension, or in worst case due to rapid chemical exchange with a contaminant proton complex. The iodine(I) complexes of tertiary amines show δ(^15^N)_coord_ of ca. −15 ppm.^109^ It is important to note that despite the halogen bonds of aliphatic amines being ∼50 kJ/mol stronger due to the larger Lewis basicity of these amines than those of the aromatic Lewis bases, their coordination shifts are significantly smaller. The large coordination shift of the pyridine complexes of halogen(I) ions is the consequence of paramagnetic ring currents (deshielding term), which are the result of the partial quaternary character of the nitrogen upon formation of the halogen bond. This is much larger than the diamagnetic shielding term, which reflects the total electron population on the nitrogen, which is similar for the halogen(I) complexes of aliphatic amines and pyridines. Hence, the larger coordination shifts of aromatic amines cannot be interpreted in terms of extra stabilization by the aromatic ring system. A further misconception occasionally found in the literature^56,108^ is that asymmetric halogen(I) complexes cannot be characterized in solution due to ligand scrambling. They are straightforwardly studied by solution NMR using bidentate ligands.^15,49^

### Computational Analyses

The geometry and energy of the halogen bond have been studied at various levels of computations (DFT, MP2, CCSD(T)).^15,19,41,45,49,54,62,77,79,80^ Benchmarking indicated that the M06 functional in combination with the aug-cc-pVTZ basis set provides the overall most accurate predictions.^110^ The LC-ωPBE, ωB97X-D, LC-TPSS, CAM-B3LYP, and B3LYP functionals also showed acceptable performance, whereas MP2, M06-HF, and HF did not. A recent study^45^ indicated that the interaction energies and the electrostatic, dispersion, and orbital terms of the interaction remain unaffected by steric hindrance. This is in agreement with related X-ray crystallographic and solution NMR observations.^111^ Energy decomposition analysis (EDA)^45^ confirms that the halogen bond of halogen(I) ions cannot be properly described by a purely electrostatic model but rather as a 3c4e bond,^18,25^ confirming the initial observations of Odd Hassel.^20^

**Figure 6:**
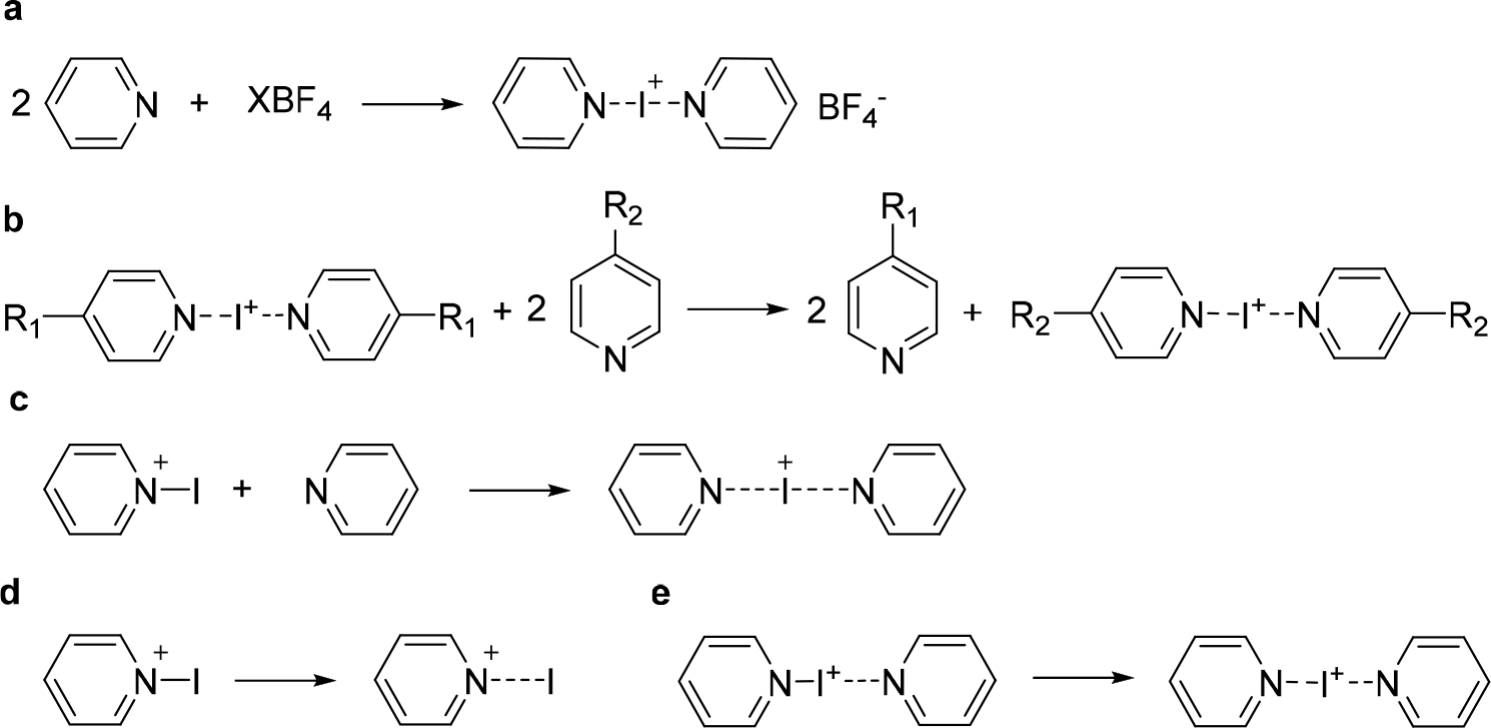
Recommended hypothetical transformations for estimation of the stability of halogen(I) complexes. Transformations for estimation of (a) the Gibbs free energy (Δ*G*) and the electronic energy (Δ*E*) of halogen(I) complexes and (b) the relative stabilities of structurally related complexes. Transformation for computational estimation of (c) the energy of asymmetric halogen(I) complexes, (d) the energy required for lengthening a D–X covalent bond to the distance corresponding to a D···X halogen bond, and (e) the energy gain/cost of forming a symmetric three-center, four-electron halogen bond from a conventional asymmetric halogen bond.

The ability to estimate bond energies and geometries for complexes that cannot be crystallized is a great advantage of computational methods. In most cases, the predicted parameters can be validated against experimental data and hence geometries against X-ray data and bond energies against kinetic observations.^77,110^ As it is an advantage to keep data originating from different laboratories comparable, we recommend standardized hypothetical reactions for the computational estimation of the energies of halogen(I) complexes using [bis(pyridine)iodine(I)]^+^ complexes as the model system for visualization (Figure 6). The transformation shown in Figure 6a may be used for the estimation of the Gibbs free energy (Δ*G*) and of the electronic energy (Δ*E*) of halogen(I) complexes, in general. Depending on the charge and thus the presence or absence of a counterion, the equation may need adjustment. The isodesmic reaction shown in Figure 6b is applicable for comparison of the relative stabilities, for instance, of close structural analogues bearing different substituents.^62,63^ The (relative) stabilities of complexes formed upon the interaction of a halogenated Lewis base with a nonhalogenated one is shown in Figure 6c. This reaction is most relevant for the estimation of the energies of the asymmetric halogen-bond complexes of, for instance, *N*-halosaccharins,^50^*N*-halosuccinimides,^112^ and hypoiodates^55,56^ as well as of fluorine-centered halogen-bond complexes.^19^ The energy cost of stretching a D–X covalent to a D···X halogen bond of a [D···X···D]^+^ complex may be calculated using the hypothetical transformation shown in Figure 6d, whereas the energy gain of forming a symmetric 3c4e complex from the corresponding asymmetric one may be calculated by applying the equation shown in Figure 6e. For estimation of the charge distribution within halogen(I) complexes, natural bond orbital analysis including natural population analysis (NPA) and second-order perturbation theory analysis of the Fox matrix is recommended; for literature examples providing further details, we direct the readers to refs (19) and (77).

## Applications

### Halogen Transfer Reactions

Halogen(I) complexes were originally introduced as mild halogen transfer agents with applications, for instance, in halofunctionalizations,^113^ halocyclizations,^114^ and alcohol oxidations.^24^ New analogues following the established concepts are still being disclosed.^115^ The progress of synthetic applications has recently been reviewed,^13^ and therefore, only some of the conceptually most important recent work is highlighted herein.

### Mechanism of Halogen(I) Transfer

**Figure 7:**
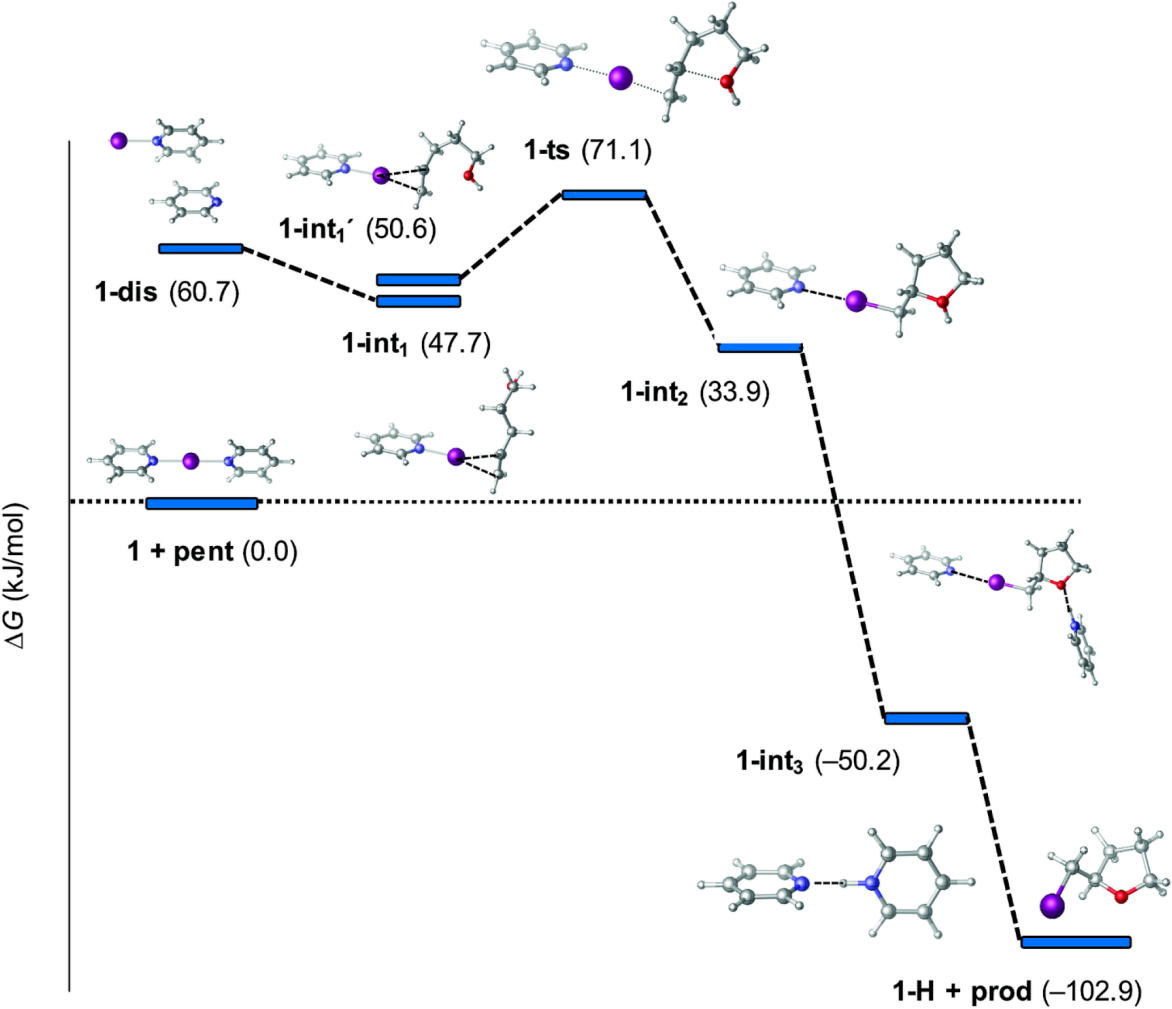
Free energy profile computed for the reaction of [bis(pyridine)iodine(I)]^+^ (**1**) with 4-penten-1-ol (**pent**) with the relative stabilities shown in parentheses (in kJ/mol) with respect to the energy of the starting materials (**1** + **pent**).^77^

Barluenga’s reagent^23,24^ has been around for a quarter of century, however, without understanding of the mechanism of the halogen(I) transfer process. The mechanism of halocyclization reactions of [bis(pyridine)iodine(I)]^+^ reagents has recently been established based on kinetic and computational (DFT) data.^77^ The free energy profile corresponding to the most feasible reaction pathway is shown in Figure 7, including the structures of the key intermediates. Iodine(I) transfer from the bidentate [1,2-bis((pyridine-2-ylethynyl)benzene)iodine(I)]^+^ complex proceeds via the same mechanism.^77^ In light of this, recent speculations suggesting that bidentate ligands may “negatively” influence the mechanism of halogen(I) transfer reactions are unsubstantiated. Based on the similarity of the bonding of halogen(I) complexes, whether symmetric (homoleptic) or asymmetric (heteroleptic), charged or neutral, encompassing aliphatic or aromatic Lewis bases with nitrogen, oxygen, or sulfur donors, the mechanism of halogen(I) transfer is similar.

### Asymmetric Halogenation

Halogen-bonded halogen(I) complexes are applicable for electrophilic halofunctionalization of the carbon–carbon double bonds of alkenes. Despite diastereoselectivity, there is no facial selectivity in such halenium additions, and accordingly, halogen(I) addition as a rule results in racemic products. The 3c4e halogen-bond complexes of chiral Lewis bases are promising candidates for the development of robust and substrate-independent enantioselective halogen transfer strategies as (i) they allow modulation of the reactivity of halogen(I) complexes via substituents of the Lewis bases while (ii) they are encompassed in a chiral environment,^62^ which are the two key challenges.

Brown, Cui, and Neverov were first to evaluate chiral monodentate pyridines for enantioselective halogen transfer reactions. They demonstrated that the complexes of these ligands do not produce an intermediate that would react with different faces of the alkenes at different rates.^117^ Halocyclizations using the halogen-bond complex of (2-substituted) pyridines lead to the formation of a [Lewis base–X]^+^ complex following the mechanism shown in Figure 7 (**1-int**_**1**_), which partitions between reversal and product formation. The relative rate of the stereoselective processes is insensitive to the nature of the substituents ortho to the donor of the Lewis base. Despite extensive efforts,^117−119^ an appreciable enantiomeric excess could not be obtained using monodentate chiral ligands. Our conclusion is that the halogen-bond complexes of monodentate ligands—independent of charge or type of donor atom—easily dissociate in solution, which leads to rapid ligand scrambling and thereby constant variation of the chiral environment. In addition, such ligands are flexible, and their chiral moieties are too far from the reaction center; hence, they are not suitable for the development of enantioselective halogenation strategies.

**Figure 8:**
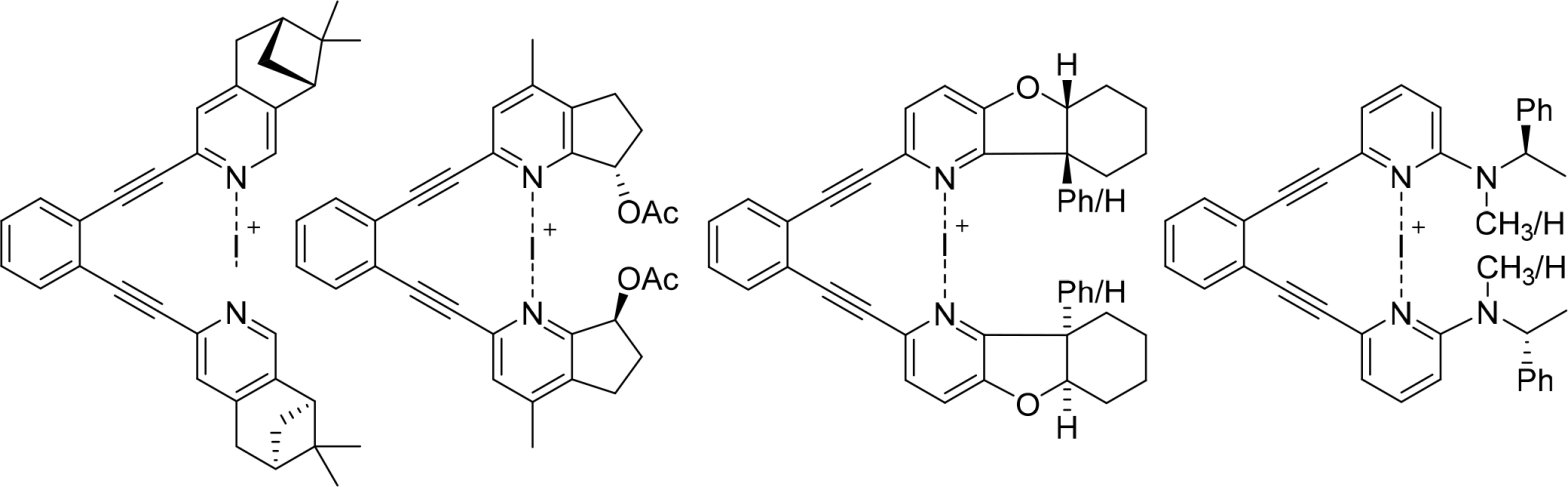
Three-center halogen-bond complexes of chiral bidentate ligands that were explored in the development of enantioselective electrophilic halofunctionalization.^116^

Chiral bidentate bis(pyridine)-type ligands were next evaluated for encapsulation of a halogen(I) ion into a chiral pocket to provide a stronger influence on the stereochemistry of halofunctionalization to make it enantioselective.^116^ Such systems (Figure 8) do not suffer from ligand scrambling and are expected to influence the halenium transfer process with both chiral substituents. The complexes shown in Figure 8 transferred iodine(I) to a model alkene, however, without significant enantioselectivity. This may be explained by insufficient substrate preorganization, by their flexibility, and by the distance between the chiral groups and the reaction center.

**Figure 9:**
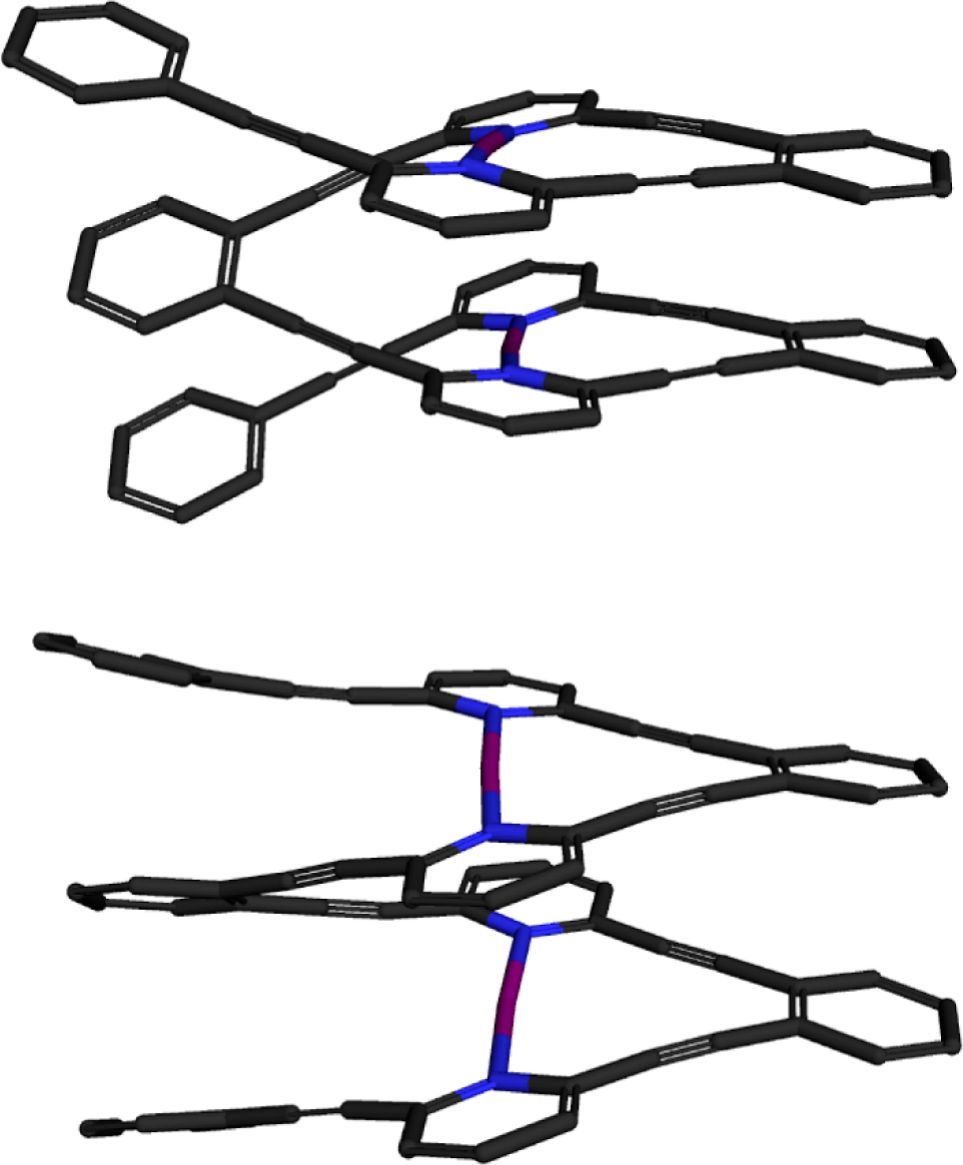
P (top) and M (bottom) stereoisomer of a halogen-bonded helicate encompassing stabilized halogen(I) ions in a chiral environment, which is stable at ambient conditions and is capable of halogen(I) transfer to alkenes.^105^ Providing a continuously chiral environment, a monochiral helix is expected to be superior for enantioselective halogen(I) transfer reactions as compared to the halogen-bond complexes of small chiral ligands shown in Figure 8.

As small chiral Lewis bases do not provide a strong enough chiral environment for enantioselective halogen(I) transfer, more advanced attempts aim at embedding halogen(I) ions into a large continuous chiral environment, such as a helicate (Figure 9).^105^ Homochiral self-sorting enantiomeric helices were prepared and shown to transfer iodine(I) to a model alkene with a controllable rate yet without enantioselectivity. This preliminary work indicated that a halogen(I) encompassing a chiral helix is stable at room temperature without the need for a protective atmosphere, whereas it is labile enough to release its halogen(I) content in the presence of an alkene. Such helices encompass a high density of halogen(I) ions that due to an efficient charge transfer from the halogen(I) ions to the conjugated backbone do not carry a full positive charge and accordingly can be packed with a remarkably short distance to each other (*d* = 1.88 Å), shorter than the sum of the van der Waals radii of two iodines (1. 98 Å) and of the sum of the cationic radii of two iodine(I) ions (2.21 Å).

The above work has triggered markedly increasing interest in the development of enantioselective halogen transfer reactants following the same strategy and using conceptually similar even if structurally different ligands (different donor atom, charge, etc.).^45,56^ Literature studies indicate that small, chiral, monodentate ligands, such as chiral pyridines and hypoiodates, are insufficient to provide significant enantiomeric excess. Instead, the design of a large continuously chiral environment, avoidance of ligand scrambling and halogen dancing (exchange of X^+^ between alkenes), rational modulation of the reactivity of halogen(I) ions, and a comprehensive understanding of the mechanism of halogen(I) transfer are necessary for success. Chiral supramolecular complexes and halogen-bonded organic frameworks currently provide the most promising strategies toward chiral halogen(I) transfer. Thorough reaction kinetics investigations are expected to be essential for progress.^62,77,117^

### Halogen(I) Catalysis

**Scheme 4:**
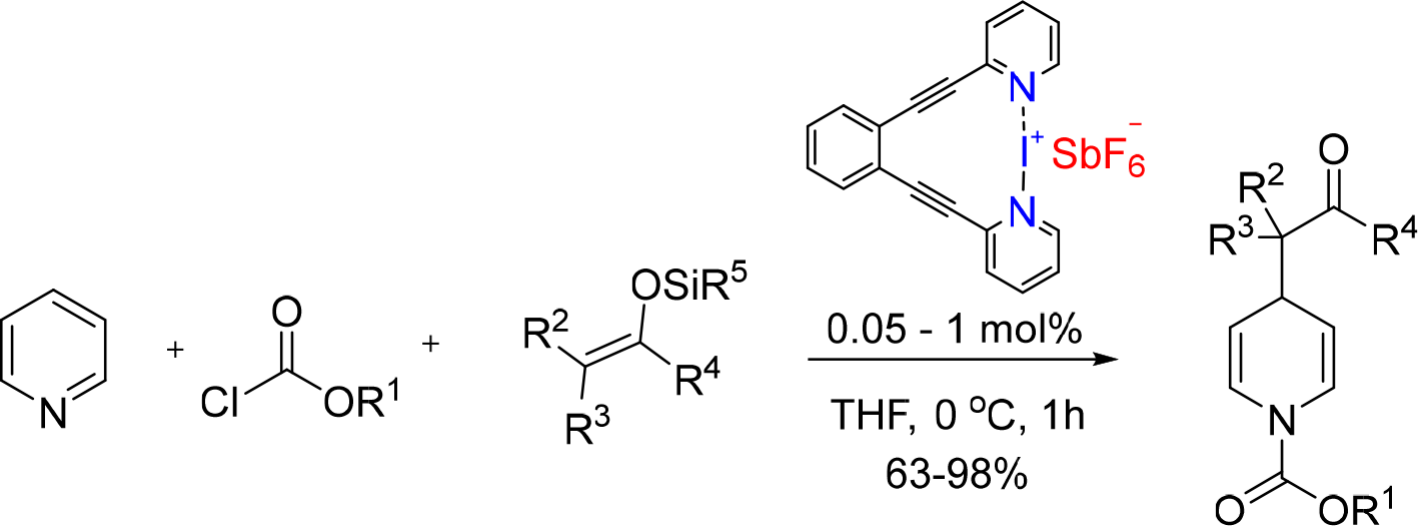
[N···I···N]^+^ Complex-Catalyzed Mukaiyama–Mannich-Type Reaction, As Proposed by Momiyama and Co-workers^120^

The iodine(I) and bromine(I) complexes of 1,2-bis(pyridine-2-ylethynyl)benzene accompanied by a non-nucleophilic counteranion have been proposed as anion binding catalysts for a Mukaiyama–Mannich-type reaction of *N*-heteroaromatics (Scheme 4) by Momiyama and co-workers,^120^ providing excellent yields even with as low as 0.1% catalyst loading. The halogen(I) of the [N···X···N]^+^ complex is suggested to participate in the exchange reactions [N···X···N]^+^ → [N···X···Cl] → [Cl···X···Cl]^−^ → [N···X···N]^+^ throughout the catalytic cycle, which involves the breakage and formation of three-center halogen-bond complexes. The role of the [N···X···N]^+^ complex in this catalytic cycle is thus the reversible release of a halogen(I) to modulate the nucleophilicity of chloride ions toward a silyl protecting group by temporarily storing them in a stabilized 3c4e [Cl···X···Cl]^+^ complex. In contrast to a series of previous applications, in which halogen(I) complexes were utilized as halogen(I) transfer agents or oxidants, this transformation makes use of the tamed reactivity of the halogen(I) ion embedded in a 3c4e halogen-bond complexes by using it as a *catalyst*. It is important to note the high stability of [N···X···N]^+^ complexes, which is true as long as they are handled under moisture-free conditions in an aprotic solvent.^121^

For further examples of the synthetic applications of halogen(I) complexes we direct the reader to the recent reviews.^13,17^

### Supramolecular Chemistry

The tamed reactivity of halogen(I) ions when embedded in halonium complexes and the strength of their halogen bonds make the [D···X···D] halogen bond into an excellent supramolecular synthon.^28,91,122^ It is highly directional and has an electron density-independent length,^44^ which makes it ideal candidate for building complex architectures. Complexes held together by several [D···I···D] interactions have been observed to be surprisingly stable and insensitive even to moisture.^88−90,105^ There is an obvious geometric similarity of iodine(I) complexes to that of silver(I), tempting one to presume that the 3D structure of silver(I) complexes can be used to extrapolate to that of analogous iodine(I) complexes. We wish to emphasize that this is not necessarily the case,^43,44^ and it thus may lead not only to unjustified presumptions but also to unfortunate misapprehensions.

Cages and capsules held together by [N···I···N]^+^ halogen bonds were reported^88−90,123,124^ to not require inert conditions for preparation and to be stable in aprotic solvents for extended periods (weeks). Some were reported to decompose upon addition of acetonitrile,^89^ most likely due to the aqueous contamination of the solvent as halogen(I) complexes are known to be stable in acetonitrile.^53,90^ Dimeric capsules were prepared utilizing the formation of four [N···X···N]^+^ bonds between ethylene-bridged tetrakis(3-pyridyl)cavitands.^123^ In contrast to the analogous silver(I)-based systems that form several types of capsules, a single type of capsule was reported for iodine(I) complexes. Using tetrakis(4-pyridyl)cavitands, octahedral hexameric capsules were assembled in chloroform whereas pentameric ones were assembled in dichloromethane.^89^ These capsules were capable of encapsulating tosylate ions. Furthermore, a symmetrical cage was obtained by the formation of [N···I···N]^+^ bonds between 1,3,5-tris(imidazole-1-ylmethyl)-2,4,6-trimethylbenzene units.^90^ Tris(1-methyl-1-azonia-4-azabicyclo[2.2.2]octane)-mesitylene, analogous in structure but bigger, formed a tetrameric [N···I···N]^+^ bond-assembled cage. An [N···I···N]^+^ bond-stabilized helicate (Figure 9) has also been reported.^105^

### Halogen-Bonded Organic Frameworks (XOF)

A recent advance has been the disclosure of two-dimensional halogen-bonded organic frameworks (XOFs) held together by [N···I···N]^+^ halogen bonds to pyridines.^57^ These mimic covalent (COF), metal (MOF), hydrogen-bonded (HOF), and supramolecular organic (SOF) frameworks. Evidently, XOFs encompassing halogen(I) ions ought to be mild and comparably stable halogen(I) transfer agents, and accordingly, they are expected to attract vast attention the coming decade.

**Figure 10:**
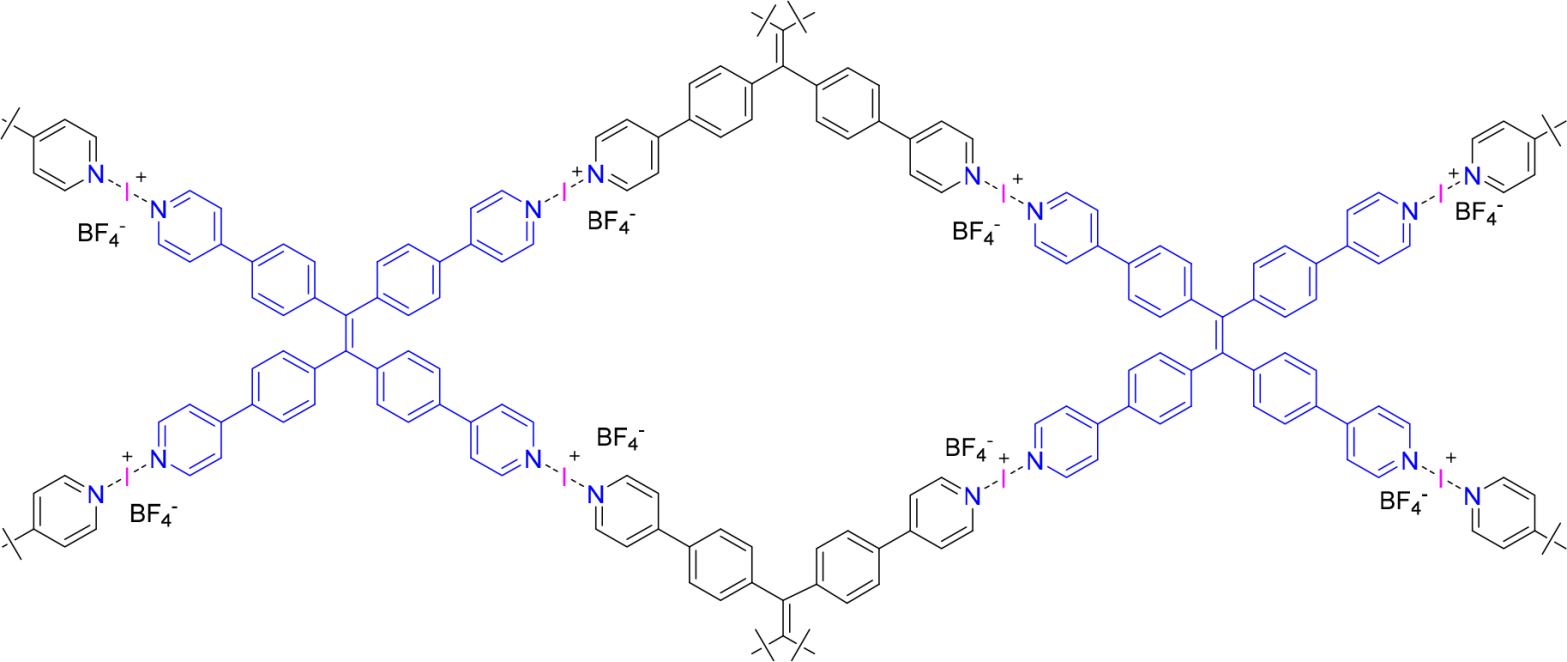
Structure of an [N···I···N]^+^ halogen-bond-bridged halogen-bonded organic framework (XOF), as proposed by Wang, Chen, and co-workers.^57^

The first XOF (Figure 10) was proposed by Wang, Chen, and co-workers, preparing it following the classical silver(I) exchange reaction using iodine.^57^ The X-ray diffractometric structure of the 4,4′-bipyridine- and pyridyl-functionalized tetraphenylethylene-based [N···Ag···N]^+^ metal organic framework has been obtained following crystallization from acetonitrile. Unfortunately, no crystal structure was obtained for the analogous iodine(I) complex. Achieving this has expectably been hindered by the presumptive [N···I···N]^+^ complex being contaminated by silver iodide coprecipitate, which forms when a silver(I) complex is converted into the corresponding iodine(I) complex (Scheme 1). The preparation of this XOF was attempted in the presence of the protic solvent methanol, further risking transformation of the iodine(I) complex into an [N···H···N]^+^ complex. The latter might be difficult to differentiate from the analogous [N···I···N]^+^ complex with techniques typically used for characterization of supramolecular frameworks, such as SAXS, SEM, TEM, PXRD, IR, and UV–vis. Subsequently, 1,3,5-tri(pyridin-4-yl)benzene- and 1,3,5-tri-4-pyridyl-1,2-ethenylbenzene-based halogen-bonded two-dimensional frameworks were presented and proposed as halogen(I) transfer agents.^125^ These complexes were prepared in a methanol–chloroform solvent mixture, with silver iodide remaining in the obtained material as coprecipitate. The obtained material was explored for use in converting aryl boronic acids into aryl iodides (39–95% yield, 80 °C, 14–20 h). This has doubtlessly been a major leap in the development of advanced iodine(I) transfer agents.

The expected next large step in the development of XOFs is the disclosure of a route that does not involve silver(I) to iodine(I) exchange as this results in unavoidable AgI and I_2_ contamination. A viable synthetic route is expected to use the exchange reaction^62^ between the [bis(pyridine)iodine(I)]^+^ complex and a multidentate ligand offering several Lewis basic sites. Following iodine(I) transfer, the byproducts may in this case easily be removed by washing with an aprotic solvent, yielding an uncontaminated XOF.

We expect a further key leap to be the first confirmation of the formation of a XOF with X-ray diffraction or MicroED instead of indirect evidence only. New types of 2D and 3D frameworks encompassing iodine(I), bromine(I), or chlorine(I) bridges and a variety of polyfunctional ligands with different donor functionalities will likely be developed.

### Functional Materials

**Figure 11:**
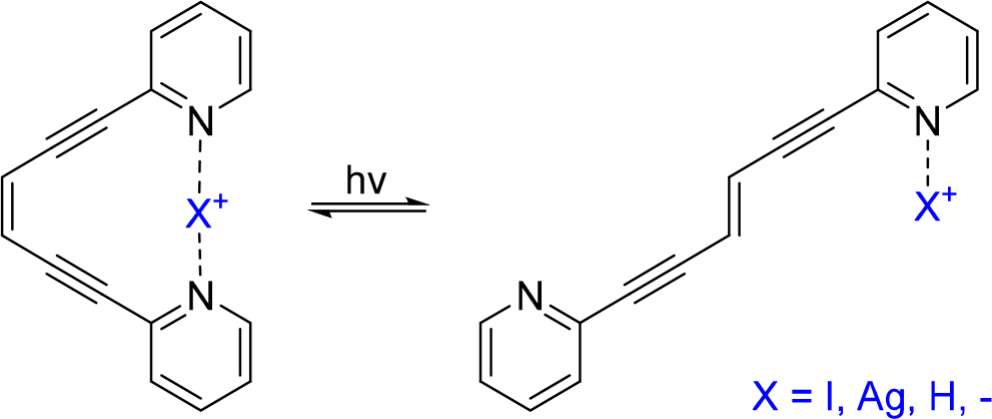
Photoswitching of an enediyne is differently modulated by a [N···I···N]^+^ halogen bond as compared to a [N···Ag···N]^+^ coordination bond or a [N···H···N]^+^ hydrogen bond.^126^ The modulation of this photoswitch foreshadows the development of a variety of halogen-bond based advanced functional materials.

The first explorations of 3c4e halogen bonds in advanced functional materials applicable in, for instance, energy conversion, mobility, cooling, medicine, and robotics have just begun. One early example demonstrated^126^ that the photoisomerization of an enediyne cis–trans switch (Figure 11) is differently modulated by a [N···I···N]^+^ halogen bond as compared to a [N···Ag···N]^+^ coordination or a [N···H···N]^+^ hydrogen bond. Hence, a [N···H···N]^+^ hydrogen bond decreases the photoisomerization rate, a coordination bond inhibits it, whereas a [N···I···N]^+^ halogen bond allows photoisomerization and is simultaneously converted to the analogous hydrogen-bonded complex.

The use of the tri(4-pyridylphenyl)amine-based [N···I···N]^+^ halogen-bonded organic framework as an absorbent of acetic and propionic acids has recently been proposed.^127^ This material has been prepared via the [N···Ag···N]^+^ to [N···I···N]^+^ exchange reaction with iodine in methanol. The resulting material absorbed acetic acid (418 mg/g, p*K*_a_ 4.8) and propionic acids (173 mg/g, p*K*_a_ 4.9) while showing negligible spectral changes (PXRD) of the framework. These vapors could be removed at 60 °C in 12 h without collapsing the framework structure. Hydrogen bonding to the anilinic amine of tri(4-pyridylphenyl)amine (∼11 kJ/mol) was proposed as the basis of the reversible absorption. Formic acid (p*K*_a_ 3.8) and trifluoroacetic acid (p*K*_a_ 0.2) induced the decomposition and dissolution of the framework. This framework is a further example of the opportunities that might be provided by three-center halogen-bond-based stimulus-responsive materials.

## Conclusions and Outlook

The past investigations of the halogen bonds of halogen(I) ions were dominated by fundamental studies aiming to reach an improved understanding of the bonding situation and the geometry and properties of halogen(I) ion complexes. Major leaps achieved by solution NMR spectroscopy were corroborated primarily by computation and X-ray diffraction and revealed that halogen(I) ions form linear 3c4e halogen bonds. Although these noncovalent bonds are unusually strong, they remain dynamic, and accordingly, [D···X···D] complexes undergo association–dissociation equilibria in solution. As the bonding situation is the same independent of the type of halogen-bond donor, the Lewis base, and the overall charge of the complex, the dynamic nature of halogen(I) complexes cannot be neglected. These complexes are stable in dry aprotic solvents, allowing their use, for instance, as organocatalysts. In the presence of moisture or protic solvents, however, they gradually decompose to their protonated [D···H···D] analogues. One should keep in mind that ligand exchange between the hydrogen-bonded, the halogen-bonded, and the analogous coordinative metal complexes may complicate the detection of such contaminants and make the differentiation between such complexes cumbersome. Halogen(I) complexes of electron donors with comparable Lewis basicity, whether mono- or bidentate, prefer a symmetric arrangement. Complexes encompassing electron donors with different Lewis basicities, whether mono- or bidentate, form asymmetric complexes, independent of the overall charge of the complex and of the Lewis base. The bond strength may be modulated by changing the electron donor ability of the Lewis bases by substituents. The change of bond strength has a minor effect only on the halogen–Lewis base interatomic distance, and it does not change the NMR coordination shifts. Halogen(I) ions are electrophilic, and their engagement in interactions as nucleophiles is questionable.

We expect that besides some further routine work confirming the already existing knowledge by complementary studies of analogues of already existing systems, the applications of the three-center halogen bonds will become the center of attention of the coming decade. Making use of the fundamental knowledge and of the experiences gained in initial investigations, we expect major steps to be taken in the development of robust enantioselective halogen transfer strategies. Small and chiral monodentate or bidentate ligands were shown to be inadequate to achieve enantiomeric access, and instead, complexes providing a large continuous chiral space are expected to lead toward success. Using halogen(I) complexes as catalysts is elegant yet may remain a curiosity with limitations in scope and applicability. Development of advanced functional materials, including XOFs and stimulus-responsive complexes, is further anticipated. Reaching major developments in materials science will require thorough structural characterization of these materials. This will help to avoid misinterpretations based on artifacts originating from contaminants in the form of synthetic byproducts and hydrogen-bonded complexes formed upon hydrolysis. We anticipate further developments in the construction of complex supramolecular architectures. Following the early examples of cages and capsules that did not have a function, apart from proving that their construction is possible, the emergence of supramolecular assemblies with cleverly engineered utilities is anticipated. Ion transporters, optoelectronic materials, organocatalysts, molecular machines, and absorbents are just a few of the countless opportunities. Undoubtedly, there will be exciting new applications with the rate of progress not being determined by the number of creative ideas and enthusiasm but rather being limited by the thoroughness of structural and mechanistic characterization and the awareness of the available fundamental knowledge.

## References

[ref1] Desiraju G. R.; Ho P. S.; Kloo L.; Legon A. C.; Marquardt R.; Metrangolo P.; Politzer P.; Resnati G.; Rissanen K. Definition of the halogen bond (IUPAC Recommendations 2013). Pure Appl. Chem. 2013, 85 (8), 1711–1713. 10.1351/PAC-REC-12-05-10.

[ref2] Cavallo G.; Metrangolo P.; Pilati T.; Resnati G.; Terraneo G. Halogen bond: a long overlooked interaction. Top. Curr. Chem. 2014, 358, 1–17. 10.1007/128_2014_573. 25504313

[ref3] Cavallo G.; Metrangolo P.; Milani R.; Pilati T.; Priimagi A.; Resnati G.; Terraneo G. The Halogen Bond. Chem. Rev. 2016, 116 (4), 2478–601. 10.1021/acs.chemrev.5b00484. 26812185 PMC4768247

[ref4] Mukherjee A.; Tothadi S.; Desiraju G. R. Halogen bonds in crystal engineering: like hydrogen bonds yet different. Acc. Chem. Res. 2014, 47 (8), 2514–2524. 10.1021/ar5001555. 25134974

[ref5] Bulfield D.; Engelage E.; Mancheski L.; Stoesser J.; Huber S. M. Crystal engineering with multipoint halogen bonding: Double two-point donors and acceptors at work. Chem. Eur. J. 2020, 26 (7), 1567–1575. 10.1002/chem.201904322. 31638284 PMC7028063

[ref6] Xu Z.; Yang Z.; Liu Y.; Lu Y.; Chen K.; Zhu W. Halogen bond: its role beyond drug-target binding affinity for drug discovery and development. J. Chem. Inf. Model. 2014, 54 (1), 69–78. 10.1021/ci400539q. 24372485

[ref7] Jiang S.; Zhang L.; Cui D.; Yao Z.; Gao B.; Lin J.; Wei D. The important role of halogen bond in substrate Selectivity of enzymatic catalysis. Sci. Rep. 2016, 6, 34750 10.1038/srep34750. 27708371 PMC5052520

[ref8] Berger G.; Soubhye J.; Meyer F. Halogen bonding in polymer science: from crystal engineering to functional supramolecular polymers and materials. Polym. Chem. 2015, 6 (19), 3559–3580. 10.1039/C5PY00354G.

[ref9] Saccone M.; Catalano L. Halogen Bonding beyond Crystals in Materials Science. J. Phys. Chem. B 2019, 123 (44), 9281–9290. 10.1021/acs.jpcb.9b07035. 31525288

[ref10] Sutar R. L.; Huber S. M. Catalysis of Organic Reactions through Halogen Bonding. ACS Catal. 2019, 9 (10), 9622–9639. 10.1021/acscatal.9b02894.

[ref11] Metrangolo P.; Neukirch H.; Pilati T.; Resnati G. Halogen bonding based recognition processes: A World parallel to hydrogen bonding. Acc. Chem. Res. 2005, 38 (5), 386–395. 10.1021/ar0400995. 15895976

[ref12] Huber S. M.; Scanlon J. D.; Jimenez-Izal E.; Ugalde J. M.; Infante I. On the directionality of halogen bonding. Phys. Chem. Chem. Phys. 2013, 15 (25), 10350–7. 10.1039/c3cp50892g. 23677285

[ref13] Turunen L.; Erdélyi M. Halogen bonds of halonium ions. Chem. Soc. Rev. 2020, 49 (9), 2688–2700. 10.1039/D0CS00034E. 32211673

[ref14] Clark T.; Hennemann M.; Murray J. S.; Politzer P. Halogen bonding: the sigma-hole. Proceedings of ″Modeling interactions in biomolecules II″, Prague, September 5th-9th, 2005. J. Mol. Model. 2007, 13 (2), 291–6. 10.1007/s00894-006-0130-2. 16927107

[ref15] Carlsson A.-C. C.; Gräfenstein J.; Budnjo A.; Laurila J. L.; Bergquist J.; Karim A.; Kleinmaier R.; Brath U.; Erdélyi M. Symmetric Halogen Bonding Is Preferred in Solution. J. Am. Chem. Soc. 2012, 134 (12), 5706–5715. 10.1021/ja301341h. 22384818

[ref16] Carlsson A.-C. C.; Gräfenstein J.; Laurila J. L.; Bergquist J.; Erdélyi M. Symmetry of [N–X–N]^+^ halogen bonds in solution. Chem. Commun. 2012, 48 (10), 1458–1460. 10.1039/C1CC15839B. 22011957

[ref17] Turunen L.; Erdelyi M. The three-center halogen bond. In Halogen Bonding in Solution; Huber S. M., Ed.; Wiley VCH: Weinheim, Germany, 2021; pp 121–151.

[ref18] Reiersolmoen A. C.; Battaglia S.; Oien-Odegaard S.; Gupta A. K.; Fiksdahl A.; Lindh R.; Erdelyi M. Symmetry of three-center, four-electron bonds. Chem. Sci. 2020, 11 (30), 7979–7990. 10.1039/D0SC02076A. 34094166 PMC8163095

[ref19] Karim A.; Reitti M.; Carlsson A.-C. C.; Gräfenstein J.; Erdélyi M. The nature of [N–Cl–N]^+^ and [N–F–N]^+^ halogen bonds in solution. Chem. Sci. 2014, 5 (8), 3226–3233. 10.1039/C4SC01175A.

[ref20] Turunen L.; Hansen J. H.; Erdelyi M. Halogen bonding: An Odd chemistry?. Chem. Rec. 2021, 21 (5), 1252–1257. 10.1002/tcr.202100060. 33939244

[ref21] Prescott A. B.; Trowbridge P. F. Periodides of pyridine. J. Am. Chem. Soc. 1895, 17, 859 10.1021/ja02166a006.

[ref22] Grimaux E. Action du brome sur la quinoleine et la pyridine. Comp. Rend. 1882, 95, 85–87.

[ref23] Barluenga J.; González J. M.; Campos P. J.; Asensio G. I(py)_2_BF_4_, a New reagent in organic synthesis: General method for the 1,2-iodofunctionalization of olefins. Angew. Chem., Int. Ed. Engl. 1985, 24 (4), 319–320. 10.1002/anie.198503191.

[ref24] Barluenga J.; González-Bobes F.; Murguía M. C.; Ananthoju S. R.; González J. M. Bis(pyridine)iodonium tetrafluoroborate (IPy_2_BF_4_): A versatile oxidizing reagent. Chem. Eur. J. 2004, 10 (17), 4206–4213. 10.1002/chem.200400136. 15352103

[ref25] Hakkert S. B.; Erdélyi M. Halogen bond symmetry: the N–X–N bond. J. Phys. Org. Chem. 2015, 28 (3), 226–233. 10.1002/poc.3325.

[ref26] Troff R. W.; Mäkelä T.; Topić F.; Valkonen A.; Raatikainen K.; Rissanen K. Alternative Motifs for Halogen Bonding. Eur. J. Org. Chem. 2013, 2013 (9), 1617–1637. 10.1002/ejoc.201201512.

[ref27] Ward J. S.; Truong K.-N.; Erdelyi M.; Rissanen K. Halogen-bonded halogen(I) ion complexes. In Comprehensive Inorganic Chemistry III; Reedijk J., Poeppelmeier K. R., Eds.; Elsevier Ltd., 2023; pp 586–601.

[ref28] Rissanen K.; Haukka M. Halonium Ions as Halogen Bond Donors in the Solid State [XL_2_]Y Complexes. In Topics in Current Chemistry; Metrangolo P., Resnati G., Eds.; Springer International Publishing: Cham, 2015; Vol. 358, pp 77–90. 10.1007/128_2014_587 25863815

[ref29] Moss G. P.; Smith P. A. S.; Tavernier D. Glossary of class names of organic compounds and reactivity intermediates based on structure (IUPAC Recommendations 1995). Pure Appl. Chem. 1995, 67, 1307–1375. 10.1351/pac199567081307.

[ref30] Roberts I.; Kimball G. E. Glossary of class names of organic compounds and reactivity intermediates based on structure (IUPAC Recommendations 1995). J. Am. Chem. Soc. 1937, 59 (5), 947–948. 10.1021/ja01284a507.

[ref31] Olah G. A.; Demember J. R. Friedel-Crafts Chemistry. 5. Isolation and Carbon-13 Nuclear Magnetic Resonance and Laser Raman Spectroscopic Study of Dimethylhalonium Fluoroantimonates. J. Am. Chem. Soc. 1970, 92 (3), 718–720. 10.1021/ja00706a058.

[ref32] Olah G. A.; Bollinger J. M. Stable Carbonium Ions. 33. Primary Alkoxycarbonium Ions. J. Am. Chem. Soc. 1967, 89 (12), 2993–2996. 10.1021/ja00988a034. 6043813

[ref33] Wieringa J. H.; Strating J.; Wynberg H. Reaction of Chlorine with Adamantylideneadamantane. Tetrahedron Lett. 1970, 11 (52), 4579–4582. 10.1016/S0040-4039(00)89422-8.

[ref34] Brown R. S.; Nagorski R. W.; Bennet A. J.; Mcclung R. E. D.; Aarts G. H. M.; Klobukowski M.; Mcdonald R.; Santarsiero B. D. Stable Bromonium and Iodonium Ions of the Hindered Olefins Adamantylideneadamantane and Bicyclo[3.3.1]Nonylidenebicyclo[3.3.1]Nonane - X-Ray Structure, Transfer of Positive Halogens to Acceptor Olefins, and Ab-Initio Studies. J. Am. Chem. Soc. 1994, 116 (6), 2448–2456. 10.1021/ja00085a027.

[ref35] Nugent W. A. Unusual Reactions of Adamantylideneadamantane with Metal Oxidants - Isolation of Stable Chloronium Salts. J. Org. Chem. 1980, 45 (22), 4533–4534. 10.1021/jo01310a064.

[ref36] Mori T.; Rathore R. X-Ray structure of bridged 2,2′-bi(adamant-2-ylidene) chloronium cation and comparison of its reactivity with a singly-bonded chloroarenium cation. Chem. Commun. 1998, (8), 927–928. 10.1039/a709063c.

[ref37] Denmark S. E.; Burk M. T.; Hoover A. J. On the Absolute Configurational Stability of Bromonium and Chloronium Ions. J. Am. Chem. Soc. 2010, 132 (4), 1232–1233. 10.1021/ja909965h. 20058922

[ref38] Rundle R. E. Probable Structure of XeF_4_ and XeF_2_. J. Am. Chem. Soc. 1963, 85 (1), 112–113. 10.1021/ja00884a026.

[ref39] Pimentel G. C. The Bonding of Trihalide and Bifluoride Ions by the Molecular Orbital Method. J. Chem. Phys. 1951, 19, 446–448. 10.1063/1.1748245.

[ref40] Rundle R. E. Electron Deficient Compounds. 2. Relative Energies of Half-Bonds. J. Chem. Phys. 1949, 17 (8), 671–675. 10.1063/1.1747367.

[ref41] Georgiou D. C.; Butler P.; Browne E. C.; Wilson D. J. D.; Dutton J. L. On the Bonding in Bis-pyridine Iodonium Cations. Aust. J. Chem. 2013, 66 (10), 1179–1188. 10.1071/CH13202.

[ref42] Koskinen L.; Hirva P.; Kalenius E.; Jääskeläinen S.; Rissanen K.; Haukka M. Halogen bonds with coordinative nature: halogen bonding in a S–I^+^–S iodonium complex. CrystEngComm 2015, 17 (6), 1231–1236. 10.1039/C4CE01735H.

[ref43] Turunen L.; Nemeth F. B.; Decato D. A.; Papai I.; Berryman O. B.; Erdelyi M. Halogen Bonds of Iodonium Ions: A World Dissimilar to Silver Coordination. B. Chem. Soc. Jpn. 2021, 94 (1), 191–196. 10.1246/bcsj.20200274.

[ref44] Bedin M.; Karim A.; Reitti M.; Carlsson A.-C. C.; Topić F.; Cetina M.; Pan F.; Havel V.; Al-Ameri F.; Sindelar V.; Rissanen K.; Gräfenstein J.; Erdelyi M. Counterion influence on the N–I–N halogen bond. Chem. Sci. 2015, 6 (7), 3746–3756. 10.1039/C5SC01053E. 29218144 PMC5707496

[ref45] Velasquez J. D.; Echeverría J.; Alvarez S. Structure and Bonding of Halonium Compounds. Inorg. Chem. 2023, 62 (23), 8980–8992. 10.1021/acs.inorgchem.3c00654. 37256722 PMC10265704

[ref46] Miyamoto K.; Uchiyama M. Hypervalent organo-λ^3^ chloranes. Chem. Lett. 2021, 50 (4), 832–838. 10.1246/cl.200849.

[ref47] Zhdankin V. V. Hypervalent iodine chemistry. Preparation, Structure and Synthetic Applications of Polyvalent Iodine Compounds; John Wiley & Sons Ltd.: Chircester, UK, 2014; p 3.

[ref48] Lin G. H. Y.; Hope H. Crystal-Structure of Bis(Thiourea)Iodine(I) Iodide. Acta Crystallogr. B Struct. Sci. 1972, 28, 643–646. 10.1107/S0567740872002900.

[ref49] Lindblad S.; Mehmeti K.; Veiga A. X.; Nekoueishahraki B.; Grafenstein J.; Erdelyi M. Halogen Bond Asymmetry in Solution. J. Am. Chem. Soc. 2018, 140 (41), 13503–13513. 10.1021/jacs.8b09467. 30234293 PMC6209183

[ref50] Yu S.; Truong K.-N.; Siepmann M.; Siiri A.; Schumacher C.; Ward J. S.; Rissanen K. Halogen-Bonded [N–I–N]^−^ Complexes with Symmetric or Asymmetric Three-Center–Four-Electron Bonds. Cryt. Growth Des. 2023, 23 (2), 662–669. 10.1021/acs.cgd.2c01162.

[ref51] Ward J. S.; Fiorini G.; Frontera A.; Rissanen K. Asymmetric [N-I-N]^+^ halonium complexes. Chem. Commun. 2020, 56 (60), 8428–8431. 10.1039/D0CC02758H. 32579654

[ref52] Chalker J. M.; Thompson A. L.; Davis B. G. Safe and Scalable Preparation of Barluenga’s Reagent. Org. Synth. 2010, 87, 288–298. 10.1002/0471264229.os087.31.

[ref53] Carlsson A.-C. C.; Uhrbom M.; Karim A.; Brath U.; Gräfenstein J.; Erdélyi M. Solvent effects on halogen bond symmetry. CrystEngComm 2013, 15 (16), 3087–3092. 10.1039/c2ce26745d.

[ref54] Lindblad S.; Németh F. B.; Földes T.; Vanderkooy A.; Pápai I.; Erdélyi M. O–I–O halogen bond of halonium ions. Chem. Commun. 2020, 56 (67), 9671–9674. 10.1039/D0CC03513K. 32696769

[ref55] Yu S.; Ward J. S.; Truong K. N.; Rissanen K. Carbonyl Hypoiodites as Extremely Strong Halogen Bond Donors. Angew. Chem., Int. Ed. Engl. 2021, 60 (38), 20739–20743. 10.1002/anie.202108126. 34268851 PMC8518949

[ref56] Mattila M.; Rissanen K.; Ward J. S. Chiral carbonyl hypoiodites. Chem. Commun. 2023, 59 (31), 4648–4651. 10.1039/D3CC00259D. 36988285

[ref57] Gong G.; Lv S.; Han J.; Xie F.; Li Q.; Xia N.; Zeng W.; Chen Y.; Wang L.; Wang J.; Chen S. Halogen-Bonded Organic Framework (XOF) Based on Iodonium-Bridged N···I+···N Interactions: A Type of Diphase Periodic Organic Network. Angew. Chem., Int. Ed. Engl. 2021, 60 (27), 14831–14835. 10.1002/anie.202102448. 33872474

[ref58] Hassel O.; Hope H. Structure of solid compound formed by addition of 2 molecules of Iodine to 1 molecule of pyridine. Acta Chem. Scand. 1961, 15 (2), 407–416. 10.3891/acta.chem.scand.15-0407.

[ref59] Aubert E.; Espinosa E.; Nicolas I.; Jeannin O.; Fourmigué M. Toward a reverse hierarchy of halogen bonding between bromine and iodine. Faraday Discuss. 2017, 203 (0), 389–406. 10.1039/C7FD00067G. 28745381

[ref60] Wilcox S.; Sethio D.; Ward J. S.; Frontera A.; Lindh R.; Rissanen K.; Erdélyi M. Do 2-coordinate iodine(i) and silver(i) complexes form nucleophilic iodonium interactions (NIIs) in solution?. Chem. Commun. 2022, 58 (32), 4977–4980. 10.1039/D2CC00994C. 35403648

[ref61] Ward J. S.; Frontera A.; Rissanen K. Retracted Article: Nucleophilic iodonium interactions (NIIs) in 2-coordinate iodine(i) and silver(i) complexes. Chem. Commun. 2021, 57 (41), 5094–5097. 10.1039/D1CC01505B. 33899860

[ref62] Carlsson A.-C. C.; Mehmeti K.; Uhrbom M.; Karim A.; Bedin M.; Puttreddy R.; Kleinmaier R.; Neverov A. A.; Nekoueishahraki B.; Gräfenstein J.; Rissanen K.; Erdélyi M. Substituent Effects on the [N–I–N]^+^ Halogen Bond. J. Am. Chem. Soc. 2016, 138 (31), 9853–9863. 10.1021/jacs.6b03842. 27265247 PMC4981895

[ref63] Rissanen K.; Ward J. S. Iodine(I) and Silver(I) Complexes Incorporating 3-Substituted Pyridines. ACS Omega 2023, 8 (26), 24064–24071. 10.1021/acsomega.3c03097. 37426204 PMC10324066

[ref64] Prohm P.; Berg W.; Rupf S. M.; Muller C.; Riedel S. On pyridine chloronium cations. Chem. Sci. 2023, 14 (9), 2325–2329. 10.1039/D2SC06757A. 36873856 PMC9977394

[ref65] von der Heiden D.; Rissanen K.; Erdélyi M. Asymmetric [N–I–N]^+^ halonium complexes in solution?. Chem. Commun. 2020, 56 (92), 14431–14434. 10.1039/D0CC06706G. 33146164

[ref66] Yu S.; Ward J. S. Ligand exchange among iodine(I) complexes. Dalton Trans. 2022, 51 (12), 4668–4674. 10.1039/D1DT04309A. 35212689

[ref67] Dutton J. L. A focus on coordination chemistry at chlorine. Chem. Sci. 2023, 14 (15), 3961–3962. 10.1039/D3SC90047A. 37063788 PMC10094295

[ref68] Nosco D. L.; Heeg M. J.; Glick M. D.; Elder R. C.; Deutsch E. Coordination stabilization of organic intermediates. Crystal structure of {[(en)_2_Co(SCH_2_CH_2_NH_2_)]_2_I}(NO_3_)_5_.4H_2_O, a stable complex of iodine(I). J. Am. Chem. Soc. 1980, 102 (26), 7784–7786. 10.1021/ja00546a028.

[ref69] Haque I.; Wood J. L. The vibrational spectra and structure of the bis(pyridine)iodine(I), bis(pyridine)bromine(I), bis(γ-picoline)iodine-(I) and bis(γ-picollne)bromine(I) cations. J. Mol. Struct. 1968, 2 (3), 217–238. 10.1016/0022-2860(68)80004-3.

[ref70] Sabin J. R. Some calculations on the lighter bis(pyridine)halogen(I) cations. J. Mol. Struct. 1972, 11 (1), 33–51. 10.1016/0022-2860(72)85220-7.

[ref71] Rubenacker G. V.; Brown T. L. Nitrogen-14 nuclear quadrupole resonance spectra of coordinated pyridine. An extended evaluation of the coordinated nitrogen model. Inorg. Chem. 1980, 19 (2), 392–398. 10.1021/ic50204a022.

[ref72] Brayer G. D.; James M. N. G. A Charge-Transfer Complex - Bis(2,4,6-trimethyl-1-pyridyl)iodonium perchlorate. Acta Crystall. B 1982, 38 (FEB), 654–657. 10.1107/S0567740882003689.

[ref73] Bigoli F.; Leporati E.; Pellinghelli M. A.; Crisponi G.; Deplane P.; Trogu E. F. Reaction of bis (morpholinoselenocarbonyl) triselenide with iodine. Existence of a 1:1 charge-transfer precursory adduct in solution in an oxidative reaction. Isolation and crystal structure of the new (N-morphoilnecarbodiselenoato)selenium(II)iodide. J. Chem. Soc., Dalton Trans. 1983, (8), 1763–1769. 10.1039/dt9830001763.

[ref74] Blair L. K.; Parris K. D.; Hii P. S.; Brock C. P. A stable bromine(+) ion complex. A twisted bicyclo[2.2.2]octane derivative. Synthesis and structure of bis(quinuclidine)bromine(I) tetrafluoroborate. J. Am. Chem. Soc. 1983, 105 (11), 3649–3653. 10.1021/ja00349a050.

[ref75] Krebs B.; Ahlers F.-P.; Lührs E. Synthese, Struktur und Eigenschaften der neuen Bromoselenate(II) [Se_3_Br_8_]^2–^, [Se_4_Br_14_]^2–^ und [Se_5_Br_12_]^2–^ Kristallstrukturen von [Cu(i-PropCN)_4_]_2_[Se_3_Br_8_], [EtPh_3_P]_2_[Se_4_Br_14_] und [n-Prop_4_N]_2_[Se_5_Br_12_]. Z. Anorg. Allg. Chem. 1991, 597 (1), 115–132. 10.1002/zaac.19915970115.

[ref76] Demartin F.; Deplano P.; Devillanova F. A.; Isaia F.; Lippolis V.; Verani G. Conductivity, FT-Raman spectra, and x-ray crystal structures of two novel [D_2_I]In (n = 3 and D = N-methylbenzothiazole-2(3H)-selone; n = 7 and D = N-methylbenzothiazole-2(3H)-thione) iodonium salts. First example of I^–^.3I_2_ heptaiodide. Inorg. Chem. 1993, 32 (17), 3694–3699. 10.1021/ic00069a025.

[ref77] Lindblad S.; Boróka Németh F.; Földes T.; von der Heiden D.; Vang H. G.; Driscoll Z. L.; Gonnering E. R.; Pápai I.; Bowling N.; Erdelyi M. The Influence of Secondary Interactions on the [N–I–N]^+^ Halogen Bond. Chem. Eur. J. 2021, 27 (55), 13748–13756. 10.1002/chem.202102575. 34339075 PMC8518683

[ref78] Ebrahimi A.; Habibi M.; Amirmijani A. The study of counterion effect on the reactivity of nucleophiles in some S_N_2 reactions in gas phase and solvent media. J. Mol. Struct. 2007, 809 (1), 115–124. 10.1016/j.theochem.2007.01.037.

[ref79] Ebrahimi A.; Razmazma H.; Samareh Delarami H. The Nature of Halogen Bonds in [N···X···N]^+^ Complexes: A Theoretical Study. Phys. Chem. Res. 2016, 4 (1), 1–15. 10.22036/PCR.2016.11619.

[ref80] Razmazma H.; Ebrahimi A. The effects of cation−π and anion−π interactions on halogen bonds in the [N···X···N]^+^ complexes: A comprehensive theoretical study. J. Mol. Graph. Model. 2018, 84, 134–144. 10.1016/j.jmgm.2018.06.006. 29975864

[ref81] Puttreddy R.; Jurček O.; Bhowmik S.; Mäkelä T.; Rissanen K. Very strong −N–X^+^···–O–N^+^ halogen bonds. Chem. Commun. 2016, 52 (11), 2338–2341. 10.1039/C5CC09487A. 26728962

[ref82] Puttreddy R.; Rautiainen J. M.; Mäkelä T.; Rissanen K. Strong N–X···O–N Halogen Bonds: A Comprehensive Study on N-Halosaccharin Pyridine N-Oxide Complexes. Angew. Chem., Int. Ed. Engl. 2019, 58 (51), 18610–18618. 10.1002/anie.201909759. 31613414

[ref83] Makhotkina O.; Lieffrig J.; Jeannin O.; Fourmigue M.; Aubert E.; Espinosa E. Cocrystal or Salt: Solid State-Controlled Iodine Shift in Crystalline Halogen-Bonded Systems. Cryt. Growth Des. 2015, 15 (7), 3464–3473. 10.1021/acs.cgd.5b00535.

[ref84] Raatikainen K.; Huuskonen J.; Rissanen K. Discriminating octahedral transition metal ions: highly selective tripodal tris-(2,2′-bipyridine) functionalized piperazine cyclophane receptor for Cu2+ ions. Dalton Trans. 2011, 40 (21), 5706–5710. 10.1039/c1dt10406c. 21505693

[ref85] Lu J.; Scheiner S. Comparison of halogen with proton transfer. Symmetric and asymmetric systems. Chem. Phys. Lett. 2019, 731, 136593 10.1016/j.cplett.2019.136593.

[ref86] Perrin C. L. Symmetry of Hydrogen Bonds: Application of NMR Method of Isotopic Perturbation and Relevance of Solvatomers. Molecules 2023, 28 (11), 4462 10.3390/molecules28114462. 37298938 PMC10254189

[ref87] Perrin C. L. Are short, low-barrier hydrogen bonds unusually strong?. Acc. Chem. Res. 2010, 43 (12), 1550–1557. 10.1021/ar100097j. 20939528

[ref88] Warzok U.; Marianski M.; Hoffmann W.; Turunen L.; Rissanen K.; Pagel K.; Schalley C. A. Surprising solvent-induced structural rearrangements in large [N···I^+^···N] halogen-bonded supramolecular capsules: an ion mobility-mass spectrometry study. Chem. Sci. 2018, 9 (44), 8343–8351. 10.1039/C8SC03040E. 30542581 PMC6243472

[ref89] Turunen L.; Warzok U.; Schalley C. A.; Rissanen K. Nano-sized I_12_L_6_ Molecular Capsules Based on the [N···I^+^···N] Halogen Bond. Chem. 2017, 3 (5), 861–869. 10.1016/j.chempr.2017.08.010.

[ref90] Turunen L.; Peuronen A.; Forsblom S.; Kalenius E.; Lahtinen M.; Rissanen K. Tetrameric and Dimeric [N···I^+^···N] Halogen-Bonded Supramolecular Cages. Chem. Eur. J. 2017, 23 (48), 11714–11718. 10.1002/chem.201702655. 28631856

[ref91] Rissanen K. Halogen bonded supramolecular complexes and networks. CrystEngComm 2008, 10 (9), 1107–1113. 10.1039/b803329n.

[ref92] Kandrnalova M.; Kokan Z.; Havel V.; Necas M.; Sindelar V. Hypervalent Iodine Based Reversible Covalent Bond in Rotaxane Synthesis. Angew. Chem., Int. Ed. Engl. 2019, 58 (50), 18182–18185. 10.1002/anie.201908953. 31587433

[ref93] Boyle P. D.; Christie J.; Dyer T.; Godfrey S. M.; Howson I. R.; McArthur C.; Omar B.; Pritchard R. G.; Williams G. R. Further structural motifs from the reactions of thioamides with diiodine and the interhalogens iodine monobromide and iodine monochloride: an FT-Raman and crystallographic study. J. Chem. Soc., Dalton Trans. 2000, 18, 3106–3112. 10.1039/b004182n.

[ref94] Corban G. J.; Hadjikakou S. K.; Hadjiliadis N.; Kubicki M.; Tiekink E. R. T.; Butler I. S.; Drougas E.; Kosmas A. M. Synthesis, Structural Characterization, and Computational Studies of Novel Diiodine Adducts with the Heterocyclic Thioamides N-Methylbenzothiazole-2-thione and Benzimidazole-2-thione: Implications with the Mechanism of Action of Antithyroid Drugs. Inorg. Chem. 2005, 44 (23), 8617–8627. 10.1021/ic0484396. 16271004

[ref95] Tamilselvi A.; Mugesh G. Interaction of heterocyclic thiols/thiones eliminated from cephalosporins with iodine and its biological implications. Bioorg. Med. Chem. Lett. 2010, 20 (12), 3692–3697. 10.1016/j.bmcl.2010.04.087. 20471830

[ref96] du Mont W.-W.; Bätcher M.; Daniliuc C.; Devillanova F. A.; Druckenbrodt C.; Jeske J.; Jones P. G.; Lippolis V.; Ruthe F.; Seppälä E. Soft–Soft Interactions Involving Iodoselenophosphonium Cations: Supramolecular Structures of Iodine Adducts of Bulky Trialkylphosphane Selenides. Eur. J. Inorg. Chem. 2008, 2008 (29), 4562–4577. 10.1002/ejic.200800513.

[ref97] Manjare S. T.; Singh H. B.; Butcher R. J. Oxidation of Carbene-Derived Selenium Diiodide with Silver Tetrafluoroborate – Isolation of Iodonium Ion Complexes with Selones. Eur. J. Org. Chem. 2013, 2013 (12), 2161–2166. 10.1002/ejic.201201238.

[ref98] de Oliveira G. M.; Faoro E.; Lang E. S. New Aryltellurenyl Iodides with Uncommon Valences: Synthetic and Structural Characteristics of [RTeTeI_2_R], [R_2_TeTeR_2_][Te_4_I_14_], and [RTe(I)I_2_] (R = 2,6-Dimethoxyphenyl). Inorg. Chem. 2009, 48 (11), 4607–4609. 10.1021/ic900193k. 19371068

[ref99] Martinez C.; Muniz K. An Iodine-Catalyzed Hofmann-Loffler Reaction. Angew. Chem., Int. Ed. Engl. 2015, 54 (28), 8287–91. 10.1002/anie.201501122. 26016458

[ref100] Muniz K.; Garcia B.; Martinez C.; Piccinelli A. Dioxoiodane Compounds as Versatile Sources for Iodine(I) Chemistry. Chemistry 2017, 23 (7), 1539–1545. 10.1002/chem.201603801. 27735101

[ref101] Guzman Santiago A. J.; Brown C. A.; Sommer R. D.; Ison E. A. Identification of key functionalization species in the Cp*Ir(III)-catalyzed-ortho halogenation of benzamides. Dalton Trans 2020, 49 (45), 16166–16174. 10.1039/D0DT00565G. 32300762

[ref102] Dolenc D.; Modec B. EDA Complexes of N-halosaccharins with N- and O-donor ligands. New J. Chem. 2009, 33 (11), 2344–2349. 10.1039/b9nj00263d.

[ref103] Ward J. S.; Frontera A.; Rissanen K. Utility of Three-Coordinate Silver Complexes Toward the Formation of Iodonium Ions. Inorg. Chem. 2021, 60 (7), 5383–5390. 10.1021/acs.inorgchem.1c00409. 33765391 PMC8154410

[ref104] Yu S. L.; Kumar P.; Ward J. S.; Frontera A.; Rissanen K. A “Nucleophilic” iodine in a halogen-bonded iodonium complex manifests an unprecedented I^+^^...^ Ag^+^ interaction. Chem. 2021, 7 (4), 948–958. 10.1016/j.chempr.2021.01.003.

[ref105] Vanderkooy A.; Gupta A. K.; Földes T.; Lindblad S.; Orthaber A.; Pápai I.; Erdélyi M. Halogen Bonding Helicates Encompassing Iodonium Cations. Angew. Chem., Int. Ed. Engl. 2019, 58 (27), 9012–9016. 10.1002/anie.201904817. 31074942 PMC6773207

[ref106] Kukkonen E.; Malinen H.; Haukka M.; Konu J. Reactivity of 4-Aminopyridine with Halogens and Interhalogens: Weak Interactions Supported Networks of 4-Aminopyridine and 4-Aminopyridinium. Cryt. Growth Des. 2019, 19 (4), 2434–2445. 10.1021/acs.cgd.9b00119.

[ref107] Suzaki Y.; Saito T.; Ide T.; Osakada K. A rhomboid-shaped organic host molecule with small binding space. Unsymmetrical and symmetrical inclusion of halonium ions. Dalton Trans. 2014, 43 (18), 6643–9. 10.1039/C3DT53629G. 24626614

[ref108] Ward J. S.; Sievanen E. I.; Rissanen K. Solid-state NMR Spectroscopy of Iodine(I) Complexes. Chem. Asian J. 2023, 18 (6), e202201203 10.1002/asia.202201203. 36734201

[ref109] Kumar P.; Komulainen J.; Frontera A.; Ward J. S.; Schalley C.; Rissanen K.; Puttreddy R. Linear bis-Coordinate Silver(I) and Iodine(I) Complexes with R_3_R_2_R_1_N Tertiary Amines. Chem. Eur. J. 2023, e202302162 10.1002/chem.202302162. 37682579

[ref110] Sethio D.; Raggi G.; Lindh R.; Erdelyi M. Halogen Bond of Halonium Ions: Benchmarking DFT Methods for the Description of NMR Chemical Shifts. J. Chem. Theory Comput. 2020, 16 (12), 7690–7701. 10.1021/acs.jctc.0c00860. 33136388 PMC7726912

[ref111] Ward J. S.; Gomila R. M.; Frontera A.; Rissanen K. Iodine(i) complexes incorporating sterically bulky 2-substituted pyridines. RSC Adv. 2022, 12 (14), 8674–8682. 10.1039/D2RA01390H. 35424827 PMC8984907

[ref112] Guzman Santiago A. J.; Brown C. A.; Sommer R. D.; Ison E. A. Identification of key functionalization species in the (CpIr)-Ir-star(III)-catalyzed-ortho halogenation of benzamides. Dalton Trans. 2020, 49 (45), 16166–16174. 10.1039/D0DT00565G. 32300762

[ref113] Barluenga J. Transferring iodine: more than a simple functional group exchange in organic synthesis. Pure Appl. Chem. 1999, 71 (3), 431–436. 10.1351/pac199971030431.

[ref114] Barluenga J.; Vazquez-Villa H.; Ballesteros A.; Gonzalez J. M. Cyclization of carbonyl groups onto alkynes upon reaction with IPy_2_BF_4_ and their trapping with nucleophiles: A versatile trigger for assembling oxygen heterocycles. J. Am. Chem. Soc. 2003, 125 (30), 9028–9029. 10.1021/ja0355372. 15369355

[ref115] Wilson L. M. E.; Rissanen K.; Ward J. S. Iodination of antipyrine with [N-I-N] and carbonyl hypoiodite iodine(i) complexes. N. J. Chem. 2023, 47 (6), 2978–2982. 10.1039/D2NJ05349G.

[ref116] von der Heiden D.; Németh F. B.; Andreasson M.; Sethio D.; Pápai I.; Erdelyi M. Are bis(pyridine)iodine(i) complexes applicable for asymmetric halogenation?. Org. Biomol. Chem. 2021, 19 (38), 8307–8323. 10.1039/D1OB01532J. 34522944 PMC8494190

[ref117] Cui X. L.; Brown R. S. Mechanistic evaluation of the halocyclization of 4-penten-1-ol by some Bis(2-substituted pyridine) and Bis(2,6-disubstituted pyridine)bromonium triflates. J. Org. Chem. 2000, 65 (18), 5653–5658. 10.1021/jo000449a. 10970306

[ref118] Brown R. S. Investigation of the early steps in electrophilic bromination through the study of the reaction with sterically encumbered olefins. Acc. Chem. Res. 1997, 30 (3), 131–137. 10.1021/ar960088e.

[ref119] Neverov A. A.; Feng H. X. M.; Hamilton K.; Brown R. S. Bis (pyridine)-based bromonium ions. Molecular structures of bis(2,4,6-collidine)bromonium perchlorate and bis(pyridine)bromonium triflate and the mechanism of the reactions of 1,2-bis(2′-pyridylethynyl)benzenebrominum triflate and bis(pyridine)bromonium triflate with acceptor olefins. J. Org. Chem. 2003, 68 (10), 3802–3810. 10.1021/jo020750m. 12737557

[ref120] Oishi S.; Fujinami T.; Masui Y.; Suzuki T.; Kato M.; Ohtsuka N.; Momiyama N. Three-center-four-electron halogen bond enables non-metallic complex catalysis for Mukaiyama-Mannich-type reaction. iScience 2022, 25 (10), 105220 10.1016/j.isci.2022.105220. 36274952 PMC9579028

[ref121] Oishi S.; Fujinami T.; Masui Y.; Suzuki T.; Kato M.; Ohtsuka N.; Momiyama N. Protocol for efficient dearomatization of N-heteroaromatics with halogen(I) complex catalyst. STAR Protoc. 2023, 4 (1), 102140 10.1016/j.xpro.2023.102140. 36892997 PMC10020682

[ref122] Aakeroy C. B.; Spartz C. L. Halogen Bonding in Supramolecular Synthesis. In Topics in Current Chemistry; Metrangolo P., Resnati G., Eds.; 2015; Vol. 358, pp 155–182. 10.1007/128_2014_567 25467531

[ref123] Turunen L.; Warzok U.; Puttreddy R.; Beyeh N. K.; Schalley C. A.; Rissanen K. [N···I^+^···N] Halogen-Bonded Dimeric Capsules from Tetrakis(3-pyridyl)ethylene Cavitands. Angew. Chem., Int. Ed. Engl. 2016, 55 (45), 14033–14036. 10.1002/anie.201607789. 27709827

[ref124] Taipale E.; Ward J. S.; Fiorini G.; Stares D. L.; Schalley C. A.; Rissanen K. Dimeric iodine(i) and silver(i) cages from tripodal N-donor ligands the [N-Ag-N] to [N-I-N] cation exchange reaction. Inorg. Chem. Front. 2022, 9 (10), 2231–2239. 10.1039/D1QI01532J.

[ref125] Xia N.; Han J.; Xie F.; Gong G.; Wang L.; Wang J.; Chen S. Construction of Halogen-Bonded Organic Frameworks (XOFs) as Novel Efficient Iodinating Agents. ACS Appl. Mater. Interfaces 2022, 14 (38), 43621–43627. 10.1021/acsami.2c11598. 36099250

[ref126] Lindblad S.; Sethio D.; Berryman O. B.; Erdelyi M. Modulating photoswitch performance with halogen, coordinative and hydrogen bonding: a comparison of relative bond strengths. Chem. Commun. 2021, 57 (51), 6261–6263. 10.1039/D1CC01827B. 34060568

[ref127] Gong G. F.; Zhao J. H.; Chen Y.; Xie F.; Lu F. H.; Wang J. K.; Wang L.; Chen S. G. An amino-type halogen-bonded organic framework for the selective adsorption of aliphatic acid vapors: insight into the competitive interactions of halogen bonds and hydrogen bonds. J. Mater. Chem. A 2022, 10 (19), 10586–10592. 10.1039/D2TA00628F.

